# Novel Plasma Metabolomic Markers Associated with Diabetes Progression in Older Puerto Ricans

**DOI:** 10.3390/metabo12060513

**Published:** 2022-06-02

**Authors:** Sona Rivas-Tumanyan, Lorena S. Pacheco, Danielle E. Haslam, Liming Liang, Katherine L. Tucker, Kaumudi J. Joshipura, Shilpa N. Bhupathiraju

**Affiliations:** 1 Office of the Assistant Dean of Research and Department of Surgical Sciences, School of Dental Medicine, University of Puerto Rico, San Juan, PR 00936, USA; sona.tumanyan@upr.edu; 2 Department of Nutrition, Harvard T. H. Chan School of Public Health, Boston, MA 02115, USA; lpacheco@hsph.harvard.edu (L.S.P.); nhdah@channing.harvard.edu (D.E.H.); 3 Channing Division of Network Medicine, Brigham and Women’s Hospital, Harvard Medical School, Boston, MA 02115, USA; 4 Departments of Epidemiology and Biostatistics, Harvard T. H. Chan School of Public Health, Boston, MA 02115, USA; lliang@hsph.harvard.edu; 5 Center for Population Health and Department of Biomedical and Nutritional Sciences, University of Massachusetts Lowell, Lowell, MA 01854, USA; katherine_tucker@uml.edu; 6 Center for Clinical Research and Health Promotion, School of Dental Medicine, University of Puerto Rico, San Juan, PR 00936, USA; kaumudi.joshipura@upr.edu; 7 Department of Epidemiology, Harvard T. H. Chan School of Public Health, Boston, MA 02115, USA

**Keywords:** diabetes, metabolites, biomarkers, Hispanic

## Abstract

We assessed longitudinal associations between plasma metabolites, their network-derived clusters, and type 2 diabetes (T2D) progression in Puerto Rican adults, a high-risk Hispanic subgroup with established health disparities. We used data from 1221 participants free of T2D and aged 40–75 years at baseline in the Boston Puerto Rican Health and San Juan Overweight Adult Longitudinal Studies. We used multivariable Poisson regression models to examine associations between baseline concentrations of metabolites and incident T2D and prediabetes. Cohort-specific estimates were combined using inverse-variance weighted fixed-effects meta-analyses. A cluster of 13 metabolites of branched chain amino acids (BCAA), and aromatic amino acid metabolism (pooled IRR = 1.87, 95% CI: 1.28; 2.73), and a cell membrane component metabolite cluster (pooled IRR = 1.54, 95% CI: 1.04; 2.27) were associated with a higher risk of incident T2D. When the metabolites were tested individually, in combined analysis, 5 metabolites involved in BCAA metabolism were associated with incident T2D. These findings highlight potential prognostic biomarkers to identify Puerto Rican adults who may be at high risk for diabetes. Future studies should examine whether diet and lifestyle can modify the associations between these metabolites and progression to T2D.

## 1. Introduction

Type 2 diabetes (T2D) is a chronic condition that affects millions of people worldwide. In 2019, T2D was the ninth leading cause of death with an estimated 1.5 million deaths [1]. Puerto Ricans have higher prevalence of T2D than non-Hispanic Whites or other Hispanic subgroups [2]. The Global Diabetes Compact [3], initiated by the World Health Organization in 2021, emphasizes the need for innovative methods to close research gaps to address this growing epidemic. Understanding the biological mechanisms of T2D development is essential for the development of targeted early diagnosis and prevention programs, especially among populations with high prevalence of T2D. Over the past decade, plasma metabolomic technologies have emerged as an innovative and powerful method to understand molecular mechanisms of T2D development [4,5]. Despite the high burden of disease, Puerto Ricans remain an understudied group, as the majority of longitudinal studies have focused on European and Asian populations [5,6].

The aim of this study was to longitudinally assess associations between individual plasma metabolites and their predefined network-derived clusters and progression to T2D in two cohorts of participants of Puerto Rican descent: (1) the San Juan Overweight Adult Longitudinal Study (SOALS), a 3-year prospective cohort of diabetes-free overweight and obese Hispanic adults in Puerto Rico, mean aged 50.6 ± 6.8 y at baseline, and (2) the Boston Puerto Rican Health Study (BPRHS), an ongoing population-based longitudinal study of Puerto Ricans adults residing in the Greater Boston area, mean age 55.4 ± 7.1 y at baseline. Together, these provided an analytic sample of 1221 participants (927 from SOALS and 294 from BPRHS).

## 2. Results

**Figure 1:**
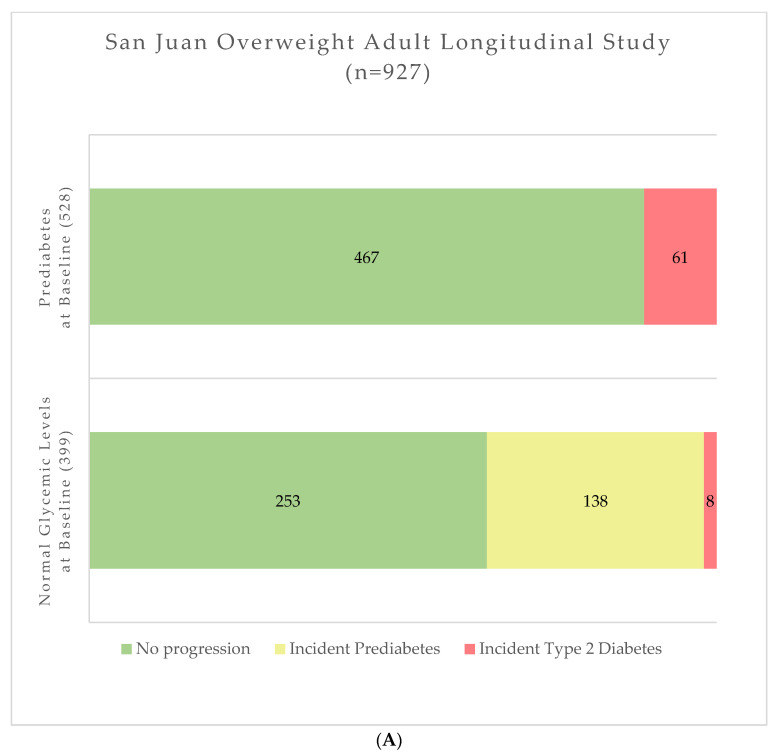

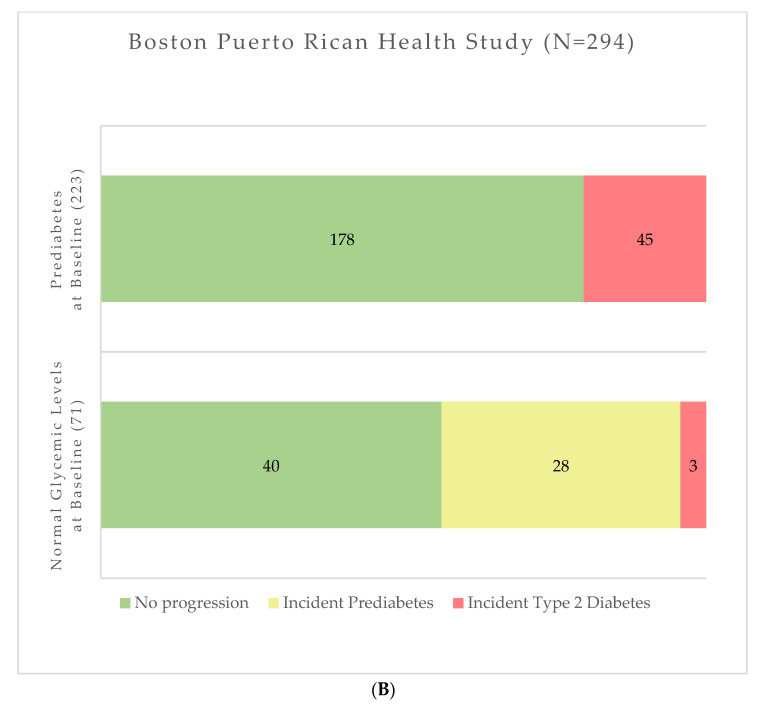
(**A**) Distribution of San Juan Overweight Adult Study (SOALS) participants at follow-up, according to baseline glycemic levels. (**B**) Distribution of Boston Puerto Rican Health Study (BPRHS) participants at follow-up, according to baseline glycemic levels.

During follow-up, a total of 69 and 48 participants progressed to T2D in SOALS (Figure 1A) and BPRHS (Figure 1B), respectively.

In SOALS, participants who did not progress to T2D were more likely to be female, have a college degree, be more physically active, and less likely to use antihypertensive medication (Table 1). In the BPRHS, participants who did not progress to T2D were more likely to be female, never smokers, alcohol consumers, to have lower waist circumference, and less likely to use antihypertensive or antilipidemic medications.

### 2.1. Progression to Type 2 Diabetes

We first examined 9 predefined metabolite clusters that were associated with prevalent T2D in a previous analysis [7] and explored their association with incident T2D among all participants and among those with prediabetes at baseline (Table 2).

A cluster consisting of 13 metabolites involved in branched chain amino acid (BCAA) and aromatic amino acid metabolism was associated with a higher risk of progression to T2D among all participants (pooled fixed-effects IRR = 1.87, 95% CI: 1.28; 2.73) and those with prediabetes at baseline (pooled fixed-effects IRR = 1.62, 95% CI: 1.09; 2.41). Another cluster, which included 8 metabolites primarily classified as cell membrane components, was associated with higher risk of progression to T2D (pooled fixed-effects IRR = 1.54, 95% CI: 1.04; 2.27). Meta-analysis findings were similar when random-effects models were considered (Supplementary Table S1). In cohort-specific analysis for this cluster, results were consistent in two cohorts (Supplementary Table S2); however, the associations were only statistically significant in the SOALS cohort. In addition to the predefined clusters, a combined cluster of 8 of BCAAs and their derivatives (leucine, isoleucine, valine, 3-methyl-2-oxovalerate, 3-methyl-2-oxobutyrate, 4-methyl-2-oxopentanoate, 2-hydroxy-3-methylvalerate, 3-hydroxyisobutyrate, identified based on individual metabolite analysis) was associated with higher risk of incident T2D, both in SOALS (IRR = 1.11; 95% CI: 1.06; 1.16) and in BPRHS (IRR = 1.07; 95% CI: 1.02; 1.14; not shown in tables). No associations were found between other clusters and T2D progression.

**Figure 2:**
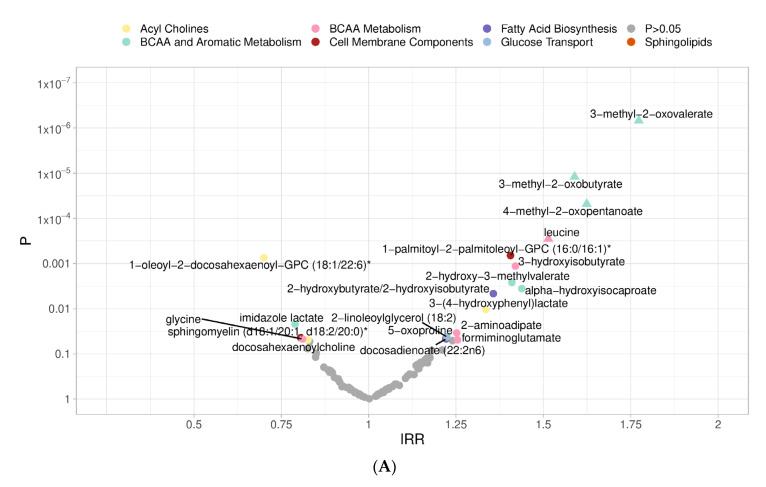

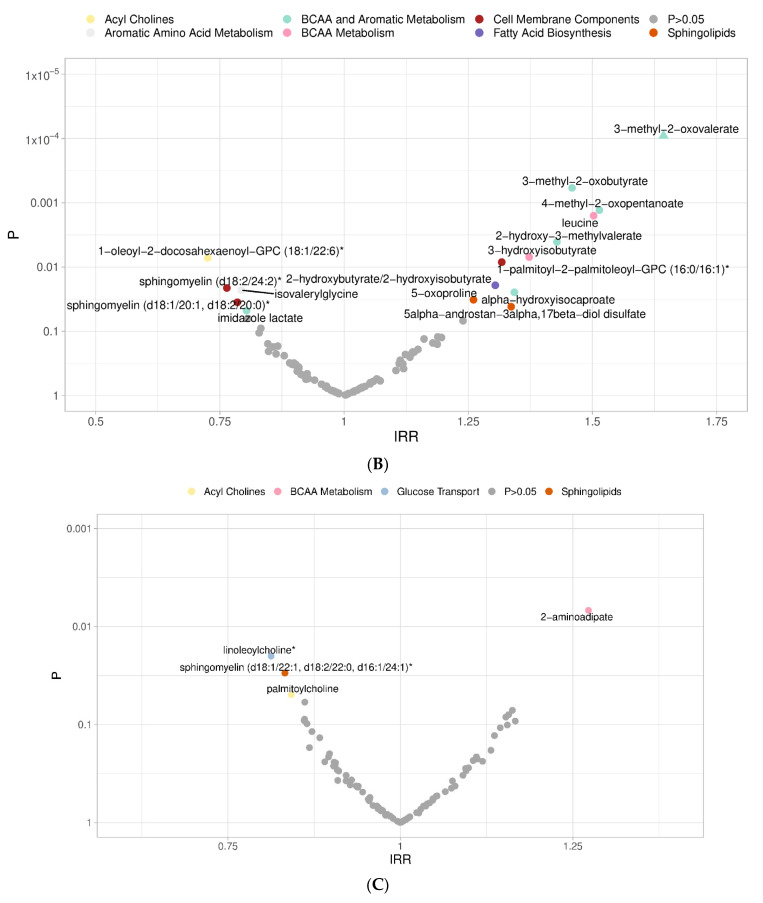

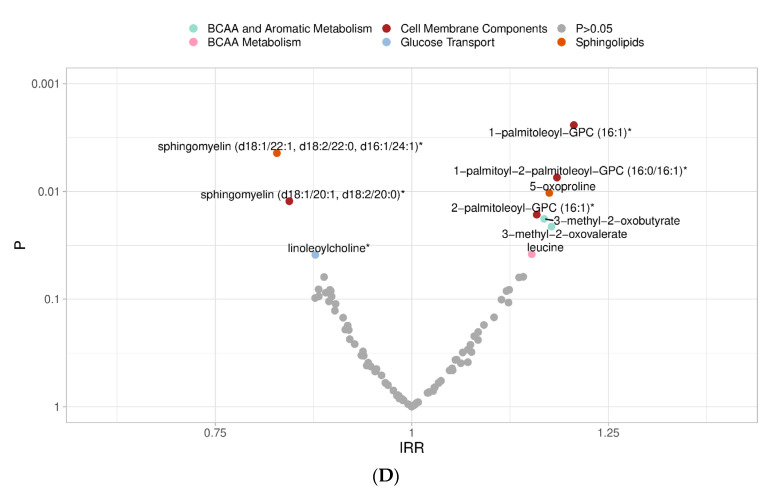
(**A**) Incidence rate ratios (IRR) and unadjusted *p*-values (P) for individual metabolites in relation to incident type 2 diabetes among all participants, according to their network-derived pathway. (**B**) Incidence rate ratios (IRR) and unadjusted *p*-values (P) for individual metabolites in relation to incident type 2 diabetes among those with prediabetes at baseline, according to their network-derived pathway. (**C**) Incidence rate ratios (IRR) and unadjusted *p*-values (P) for individual metabolites in relation to incident prediabetes among participants with normal glycemic levels at baseline, according to their network-derived pathway. (**D**) Incidence rate ratios (IRR) and *p*-values (P) for individual metabolites in relation to incident prediabetes or type 2 diabetes among all participants, according to their network-derived pathway. Metabolites with unadjusted *p*-values < 0.05 are colored according to their network-derived cluster membership; metabolites with FDR-adjusted *p*-values < 0.05 are indicated with a triangle-shaped marker.

Next, regression analyses were conducted to examine the associations between 91 individual metabolites within the clusters and 13 additional candidate metabolites. In the meta-analysis, after Benjamini–Hochberg False Discovery Rate (FDR) correction for multiple testing, only 5 metabolites were found to have statistically significant association with incident T2D: 3-methyl-2-oxovalerate (pooled IRR = 1.77, 95% CI: 1.41; 2.22), 3-methyl-2-oxobutyrate (pooled IRR = 1.59, 95% CI: 1.29; 1.96), 4-methyl-2-oxopentanoate (pooled IRR = 1.62; 95% CI: 1.29; 2.05), isoleucine (pooled IRR = 1.51, 95% CI: 1.23; 1.87) and leucine (pooled IRR: 1.51, 95% CI: 1.21; 1.89) (Figure 2A).

In the SOALS cohort, 15 metabolites showed statistically significant associations (FDR < 0.05) with progression to T2Ds (Supplementary Table S3), including 8 metabolites participating in BCAA (leucine, isoleucine, and valine) metabolism pathway (3-methyl-2-oxovalerate, 3-methyl-2-oxobutyrate, 4-methyl-2-oxopentanoate, 2-hydroxy-3-methylvalerate, isoleucine, 3-hydroxyisobutyrate, leucine, valine). In the BPRHS, we did not observe a significant association between metabolites and progression to T2D (Supplementary Table S4).

### 2.2. Progression from Prediabetes to Type 2 Diabetes

When the T2D progression analysis was repeated among 528 SOALS participants with prediabetes at baseline, 61 were identified as having T2D at follow-up (Figure 1A). In the BPRHS, among 223 participants with prediabetes at baseline, 45 were identified as having T2D at follow-up (Figure 1B). Similar to findings in the overall cohort, a cluster comprising of metabolites in the BCAA (leucine, isoleucine, valine) and aromatic amino acid (tyrosine, phenylalanine, tryptophan) metabolism (pooled IRR = 1.62, 95% CI: 1.09; 2.41), as well as the cluster formed only with 8 metabolites involved in the BCAA pathway were significantly associated with higher risk of progression from prediabetes to T2D (pooled IRR = 1.09, 95% CI: 1.04; 1.13; not shown in tables). Cohort-specific results of this subgroup analysis are presented in Supplementary Table S2. In individual metabolite analysis (Figure 2B), after FDR adjustment only the association between 3-methyl-2-oxovalerate reached statistical significance (pooled IRR= 1.64; 95% CI: 1.28; 2.11).

In cohort-specific analysis, 7 metabolites were identified in SOALS, all of which showed significant associations in the overall group (Supplementary Table S5).

### 2.3. Progression of Normoglycemic Participants to Prediabetes

In the subgroup of 399 SOALS participants with normal blood glucose levels at baseline, 8 participants experienced progression to T2D (and therefore were excluded from analysis on progression to prediabetes), and 138 progressed to prediabetes at follow-up (Figure 1A). In the BPRHS, out of 71 participants with normal glycemia at baseline, 3 participants progressed to T2D and 28 progressed to prediabetes at follow-up (Figure 1B). The pooled IRR estimates for all metabolite clusters were not statistically significant (Table 2). The metabolite cluster analysis in SOALS (Supplementary Table S2) showed a significant inverse association between a cluster of 9 metabolites in acyl choline metabolism pathway (IRR = 0.72; 95% CI: 0.53; 0.98) and a cluster of 6 metabolites involved in glucose transport (IRR = 0.68, 95% CI: 0.48; 0.95). These results, however, were not replicated in BPRHS. When metabolites were tested individually, after adjusting for multiple comparisons, no associations reached statistical significance (pooled results are presented in Figure 2C).

### 2.4. Progression to Prediabetes or Type 2 Diabetes

Among all SOALS participants, 207 participants experienced progression (Figure 1A: 138 progressed to prediabetes; 69 progressed to T2D). Among all BPRHS participants, 76 participants experienced progression (Figure 1B: 28 progressed to prediabetes; 48 progressed to T2D). In the analysis on this secondary outcome, no predefined clusters of metabolites showed significant associations with progression (Table 2). When results from individual metabolites were pooled across two cohorts, none of the metabolites achieved statistically significant associations after adjusting for multiple testing (Figure 2D).

## 3. Discussion

The current study employed a targeted approach to identify metabolites that are associated with the risk of incident T2D and prediabetes in two cohorts drawn from populations of Puerto Rican descent. The candidate 104 metabolites and their clusters included those involved in protein, lipid and carbohydrate metabolism, previously reported in the literature, and identified in cross-sectional analysis in the same cohorts: SOALS and BPRHS [7]. In the current analysis, three different outcomes were considered (incident T2D, incident prediabetes, or any progression), which allowed us to explore the role of these novel biomarkers in each step of development of the metabolic condition. In cluster analysis, the cluster formed as a weighted sum of 13 metabolites of BCAA, and aromatic amino acid metabolism was associated with higher risk of T2D among all participants (in SOALS separately and in meta-analysis, with pooled IRR = 1.87, 95% CI: 1.28; 2.73). When the analysis was limited to those with prediabetes at baseline, the BCAA/aromatic amino acid metabolism-related cluster remained strongly associated with T2D risk (pooled IRR = 1.62, 95% CI: 1.09; 2.41). Another cluster of 8 cell membrane component metabolites was associated with increased risk of incident T2D (pooled IRR = 1.54, 95% CI: 1.04; 2.27).

In individual metabolite analysis in the SOALS cohort, we identified 15 metabolites associated with incident T2D with high statistical significance. Findings from the confirmation cohort (BPRHS) were similar, but did not reach statistical significance, possibly due to a substantially smaller sample size compared to SOALS.

Meta-analysis of data from the two cohorts showed 5 metabolites (3-methyl-2-oxovalerate, 3-methyl-2-oxobutyrate, 4-methyl-2-oxopentanoate, isoleucine and leucine) that were associated with higher risk of incident T2D.

Eight of the 15 metabolites that were associated with a higher risk of incident T2D in SOALS were BCAAs, and their derivative branched-chain keto acids (BCKAs): leucine, isoleucine, valine, 3-methyl-2-oxovalerate, 3-methyl-2-oxobutyrate, 4-methyl-2-oxopentanoate, 2-hydroxy-3-methylvalerate, 3-hydroxyisobutyrate. Over the past decade, numerous prospective studies have shown significant associations between BCAAs and incident diabetes and prediabetes, with the majority of the studies coming from Caucasian and Asian population cohorts [5,6]. The recent meta-analysis of data from prospective studies on metabolites and diabetes risk [5] reported pooled relative risks that were similar to those found in our study: 1.54 (95% CI: 1.36; 1.74) for isoleucine, 1.40 (95% CI: 1.29; 1.52) for leucine, and 1.40 (95% CI: 1.25; 1.57) for valine. The current study is the first one to confirm this association longitudinally in two cohorts of Puerto Ricans, a population considered to be a high-risk group for T2D. In addition to analysis of individual BCAAs and BCKAs and the cluster of 13 BCAA/aromatic amino acid metabolism-related metabolites, a combined cluster of 8 of these metabolites was associated with an increase in T2D risk both in SOALS (IRR = 1.11; 95% CI: 1.06; 1.16) and in BPRHS (IRR = 1.08; 1.02; 1.14).

While the longitudinal associations between the 3 BCAAs (leucine, isoleucine and valine) and incident T2D have been addressed before [8], only a few studies have explored associations between BCKAs and T2D and other metabolic outcomes. To our knowledge, a recent nested case-control study in a Swedish population [9] was the only prospective study to date to report an association between 3-methyl-2-oxovalerate and incident T2D, with results similar to those found in our cohorts: the OR for 1 SD increase was 1.90 (95% CI: 1.33; 2.51) in multivariable analysis. A few case-control studies included 3-methyl-2-oxovalerate [10,11], 3-methyl-2-oxobutyrate [10,11] and 4-methyl-2-oxopentanoate [10,12], reporting higher odds of T2D with higher BCKAs.

BCAAs are essential amino acids, and protein-rich foods that contain BCAAs (such as poultry, fish, lentils and beans, etc.) are known for their beneficial effects on metabolism. However, increases in fasting circulating BCAAs, and especially their toxic derivative BCKAs, may be indicative of impaired BCAA metabolism (BCAA dysmetabolism) [13], rather than greater dietary consumption. Impaired BCAA metabolism and accumulation of BCKAs and metabolites has been previously hypothesized to contribute to insulin resistance [14]. In baseline cross-sectional analysis of our study cohorts, the BCAA and aromatic amino acid cluster was previously shown to be significantly associated with higher triglyceride concentrations in both cohorts, as well as with higher glucose, insulin, HOMA-IR and HbA1c in SOALS [7].

In individual analysis of metabolites of cell membrane components, sphingomyelins were inversely associated with T2D risk in SOALS, consistent with findings from the cross-sectional analysis [7]. On the other hand, 1-Palmitoyl-2-palmitoleoyl-sn-glycero-3-phosphocholine (16:0/16:1), otherwise known as phosphatidylcholine (PC) (16:0/16:1), was significantly associated with higher risk of T2D (pooled IRR = 1.41, 95% CI: 1.16; 1.71), with the overall cluster of cell membrane components showing a significant association with higher risk in SOALS, as well as in meta-analysis of two cohorts (pooled IRR = 1.54, 95% CI: 1.04, 2.27). In a recent analysis of a cohort of middle-aged and older Chinese [15], PC (16:0/16:1) was associated with diabetes, as well as an unhealthy dietary pattern that was high in refined grains and low in fish, dairy and soy products. Studies on associations between sphingolipids and incident T2D have produced mixed results [16,17,18,19,20,21,22], with recent analyses focusing on the possible differential effects of saturated vs. unsaturated sphinoglipids [22]. Similar to our findings in SOALS, analysis of sphingolipids in the Hispanic Community Health Study / Study of Latinos (HCHS/SOL) revealed an inverse association between the unsaturated sphingomyelin (d18:2/24:2) and incident diabetes, which, however, did not reach statistical significance after adjusting for multiple testing [22]. Further analysis of these metabolites in relation to dietary patterns, as well as other cardiometabolic risk factors, will elucidate potential biological mechanisms playing a role in progression to T2D.

Although metabolites involved in glutathione metabolism have been previously identified as markers of diabetes [23], our analysis in SOALS was, to our knowledge, the first longitudinal analysis to suggest that 5-oxoproline, an intermediate product in glutathione metabolism [24], may be associated with the risk of incident T2D. One standard deviation increase in 5-oxoproline was associated with a 50% higher risk of T2D (IRR in SOALS: 1.50, 95% CI: 1.17; 1.93); the association remained significant when the analysis was limited to participants identified as having prediabetes at baseline (IRR = 1.62, 95% CI: 1.22; 2.15). The analysis in our replication cohort (BPRHS), however, did not demonstrate similar results, and consideration of this metabolite in other cohorts is important in planning for future studies.

Another novel marker identified in our analysis in relation to progression to T2D was ornithine. In a recent analysis of the Singapore Prospective Study Program [25], ornithine showed significant, but modest, association with risk of T2D (IRR = 1.20, 95% CI: 1.07; 1.34). In our analysis, the association between ornithine and T2D risk were stronger (IRR in SOALS: 1.47, 95% CI: 1.14; 1.90) and highly significant. Ornithine is produced during the urea cycle; an increase in arginase activity may result in higher ornithine level and decreased nitric oxide bioavailability and can lead to metabolic complications including diabetes [26]. In a recent cross-sectional evaluation of Chinese adults, however, ornithine was inversely associated with T2D risk [27]: with OR = 0.89 (95% CI: 0.88; 0.91), similar to results obtained in our replication cohort (IRR in BPRHS: 0.71, 95% CI: 0.50; 1.03). Further exploration of the associations between this metabolite and diabetes outcomes, as well as other cardiometabolic risk factors, will contribute to a better understanding of the role of ornithine in diabetes.

Our study has several strengths. This is the first study to examine metabolites associated with T2D progression in participants of Puerto Rican descent, a population with a high prevalence of T2D. Second, the longitudinal design of both studies allowed us to look at incident outcomes. Third, detailed data collection on several confounders allowed us to control for numerous diabetes risk factors in our multivariable analysis. Finally, our analysis was unique in that it focused on different progression outcomes, allowing for potential differences in the effect of metabolites and their clusters on each stage of diabetes development. Our findings need to be interpreted in the context of a few limitations. We were limited in statistical power for some outcomes. In a cohort-specific analysis, our results appeared to be stronger for SOALS participants, compared to the replication BPRHS cohort. This discrepancy may be due to a larger sample size in the SOALS analysis (for all study outcomes), as well as methodological differences between the cohorts.

## 4. Materials and Methods

### 4.1. Study Cohorts

SOALS was designed as a three-year prospective cohort aiming to assess the bi-directional association between periodontitis and T2D. Study participant recruitment and data collection methods have been published elsewhere [28]. Briefly, this study included middle-aged (40–65 years), overweight and obese adults free from previous diagnosis of diabetes, who underwent baseline and follow-up examinations. The study data collection methods included interviewer-administered questionnaires, anthropometric measurements, as well as assessments of fasting, 30, 60, and 120 min post-load glucose and hemoglobin A1c (HbA1c).

BPRHS is an ongoing population-based longitudinal study of 1500 Puerto Ricans adults aged 45–75 years, residing in the Greater Boston area. The BPRHS was designed to examine the role of psychosocial stress on the presence and development of allostatic load and health outcomes in Puerto Ricans. Recruitment and data collection methods have been previously published [29]. Participants were recruited using door-to-door enumeration from areas of high Hispanic density, and by community outreach strategies including advertisement and community events. Study home visits occurred at baseline, 2 years, and 5 years, by bilingual interviewers.

### 4.2. Assessment of Plasma Metabolite Levels

Fasting blood samples were collected at baseline from participants in both cohorts. In SOALS, the samples were drawn in a laboratory during the baseline visit, processed, and stored at −80 °C. In BPRHS, blood samples were drawn in participants’ homes, spun using a portable centrifuge, and transported to the laboratory on dry ice, processed and stored at −70 °C. Blinded plasma specimens were assayed by Metabolon software (Durham, NC, USA), as previously described [30]. Chemical peaks were identified using positive and negative ionization ultra-high-performance liquid chromatograph–tandem mass spectrometry and gas chromatography–mass spectrometry. Metabolon software was used to identify the known metabolites; internal quality control samples were also included and injection order was random. Details describing the construction of a global metabolite network and detection of clusters within the global network have been previously described [7]. Briefly, metabolites detected in both the BPRHS and SOALS cohorts with low missingness (detection rate < 75%) were utilized to generate the global metabolite network. Undetectable values were imputed at a value equal to half the minimum of each measured metabolite. All metabolite levels were transformed using inverse normal transformation [7]. Edges and paths significantly correlated in both cohorts were retained in the final global network. An established greedy optimization algorithm [31] was utilized to identify clusters within the global network. In this analysis, we focus on metabolites within 9 clusters that were significantly associated with prevalent T2D among BPRHS and SOALS participants in this previous study: clusters of (1) 18 sphingolipids, (2) 15 metabolites of BCAA metabolism, (3) 13 metabolites of BCAA and aromatic amino acid metabolism, (4) 9 metabolites of acyl choline metabolism, (5) 11 metabolites of aromatic amino acid metabolism, (6) 8 metabolites of cell membrane components, (7) 6 metabolites of glucose transport, (8) metabolites of fatty acid biosynthesis, and (9) metabolites of sugar metabolism (the list of specific metabolites in each cluster is included in Table 2). We additionally examined 13 metabolites that have been associated with T2D in previous studies: isoleucine [5,32,33,34], valine [5,32,33,34], tyrosine [5], ornithine [25,27], betaine [5], trimethylamine N-oxide [35], carnitine, phenylalanine [5], fumarate [36], citrulline [34], aspartate [37], choline [38], and 3-aminoisobutyrate [39].

### 4.3. Assessment of Study Outcomes

In SOALS, participants were classified as having T2D at baseline based on fasting glucose ≥ 126 mg/dL, 2 h post-load glucose ≥ 200 mg/dL, or HbA1c ≥ 6.5%. During the follow-up, participants were considered as having T2D if they reported a diagnosis by a physician, or met the criteria described above. Participants were considered as having prediabetes if fasting glucose was measured between 100 and 125 mg/dL, 2 h post-load glucose was 140–199 mb/dL, or HbA1c was 5.7–6.4%. In the BPRHS, T2D was defined as: fasting glucose ≥ 126 mg/dL, or HbA1c ≥ 6.5%, or reporting use of blood-sugar lowering medication. BPRHS participants were classified as having prediabetes if their fasting glucose was between 100 and 125 mg/dL, or HbA1c was between 5.7 and 6.4%. In both cohorts, if the participants did not meet the prediabetes or T2D criteria, they were classified as having normal blood glucose.

Incident T2D diagnosed at the follow-up examination was the primary outcome of this study. Secondary outcomes were also considered: progression to prediabetes among those with normal baseline blood glucose levels, as well as any progression throughout the follow-up (from a normoglycemic to prediabetes or T2D status; and from prediabetes to T2D status).

### 4.4. Assessment of Potential Confounders

Information on potential confounders, such as age, sex, smoking, education, family history of diabetes (not measured in BPRHS), alcohol consumption and medication use was self-reported by participants during baseline visits in both cohorts, using interviewer-administered questionnaires. Anthropometric measurements, including height, weight, and waist circumference were taken 2–3 times using standard methods by trained interviewers, and the average measure was used. In SOALS, metabolic equivalent score (MET) was calculated based on self-reported time and frequency of physical activities during a typical week. In BPRHS, the physical activity score was assessed using a modified version of the Paffenbarger questionnaire [40,41].

### 4.5. Data Analysis Methods

SOALS participants with data from baseline and follow-up examinations (N = 1028) were included in the analysis. Participants were further excluded if they had diabetes at baseline (*n* = 77), had missing information on diabetes status at the second visit (*n* = 44), or missing information on physical activity (*n* = 1) or alcohol consumption (*n* = 7), or did not have available metabolic data (*n* = 17), resulting in the final sample size of 927 participants for this cohort.

BPRHS participants with data from baseline and follow-up examinations (*n* = 604) were included in the analysis. Participants were further excluded if they had diabetes at baseline (*n* = 310), had missing information on diabetes status at follow-up (*n* = 0), or missing information on physical activity (*n* = 0) or alcohol consumption (*n* = 0), or did not have available metabolic data (*n* = 0), resulting in the final sample size of 294 participants for this cohort.

We used a targeted approach to the selection of candidate metabolites, based on the literature and our previous findings from the cross-sectional analysis of the BPRHS participants [7]. Nine metabolic clusters from our previous analysis and normalized levels of 91 individual metabolites within these clusters and 13 literature-derived metabolites were considered [7]. An additional cluster of 8 metabolites involved in the isoleucine, leucine and valine pathway (formed as the sum of normalized levels of 3-methyl-2-oxovalerate, 3-methyl-2-oxobutyrate, 4-methyl-2-oxopentanoate, 2-hydroxy-3-methylvalerate, isoleucine, 3-hydroxyisobutyrate, leucine, and valine) was also explored in relation to the study outcomes.

We used Poisson regression models with log-transformed follow-up time as the offset variable and adjusted for age (years) and sex. In multivariable model 1, we adjusted for smoking (current, former, never); education (less than high school, high school, some college, college degree) in SOALS, and education (less than high school, high school, and some college or more) in BPRHS; family history of diabetes (yes/no) only in SOALS; physical activity measured in METs in SOALS, and physical activity score in BPRHS; waist circumference (cm), BMI (kg/m^2^), and alcohol consumption (g/day). Multivariable model 2 additionally included use of antihypertensive medications (yes/no) and statins or other lipid-lowering medications (yes/no). Cohort-specific estimates were combined using an inverse-variance weighted fixed-effects meta-analysis within the R package meta, developed by Guido Schwarzer [42,43,44].

For the analysis on progression to T2D, the regression models were repeated among 528 and 223 participants who were identified as having prediabetes at baseline for SOALS, and BPRHS, respectively. Analysis on the secondary outcome of progression to prediabetes was conducted among 399 and 71 participants with normal blood glucose concentration at baseline in SOALS and BPRHS, respectively.

*p*-values obtained from regression models on 104 individual metabolites were adjusted for multiple comparisons using the Benjamini–Hochberg FDR method [45]; a 0.05 level of statistical significance was used for all analyses. Incidence rate ratios (95% confidence intervals) for 1 standard deviation change in normalized levels of metabolites were reported. All regression analysis was conducted using SAS statistical software version 9.4 (SAS Institute, Cary, NC, USA); meta-analysis was conducted using R statistical package (V.4.1.3, R Core Team, Vienna, Austria) [42,43,44].

## 5. Conclusions

In summary, our study demonstrates that markers of BCAA and aromatic amino acid metabolism, as well as cell membrane metabolites, may play important roles in T2D development among Puerto Rican adults. Our findings highlight potential prognostic biomarkers to identify Puerto Rican adults who may be at high risk for progression to T2D. Future studies should examine whether diet and lifestyle can modify the associations between these metabolites and diabetes risk.

## Figures and Tables

**Table 1:** Distribution of SOALS and BPRHS participant characteristics, among all, and by type 2 diabetes progression status.

Characteristic	SOALS			BPRHS		
	All (*n* = 927)	Participants Who Progressed to Type 2 Diabetes (*n* = 69)	Participants Who Did Not Progress to Type 2 Diabetes (*n* = 858)	All (*n* = 294)	Participants Who Progressed to Type 2 Diabetes (*n* = 48)	Participants Who Did Not Progress to Type 2 Diabetes (*n* = 246)
Age, years						
Mean ± SD	50.6 ± 6.8	50.5 ± 6.6	50.6 ± 6.8	55.4 ± 7.1	56.1 ± 7.1	55.3 ± 7.2
Median (IQR)	50.0 (45.0–56.0)	50.0 (46.0–55.0)	50.0 (45.0–56.0)	54.0 (50.0–61.0)	54.5 (50.5–61.0)	54.0 (50.0–60.0)
Biological sex, male, n (%)	239 (25.8)	25 (36.2)	214 (24.9)	73 (24.8)	13 (27.1)	60 (24.4)
Smoking history						
Current	168 (18.1)	17 (24.6)	151 (17.6)	77 (26.2)	10 (20.8)	67 (27.2)
Former	165 (17.8)	14 (20.3)	151 (17.6)	82 (27.9)	19 (39.6)	63 (25.6)
Never	594 (64.1)	38 (55.1)	556 (64.8)	135 (45.9)	19 (39.6)	116 (47.2)
Family history of diabetes, yes, n (%)	572 (61.7)	43 (62.3)	529 (61.7)	-	-	-
Educational level ^a^, n (%)						
Less than high school	101 (10.9)	6 (8.7)	95 (11.1)	139 (47.3)	27 (56.3)	112 (45.5)
High school	399 (43.0)	29 (42.0)	370 (43.1)	112 (38.1)	14 (29.2)	98 (39.8)
Some college	130 (14.0)	15 (21.7)	115 (13.4)	43 (14.6)	7 (14.6)	36 (14.6)
College degree	297 (32.0)	19 (27.5)	370 (43.1)			
Physical activity ^b^, METs						
Mean ± SD	22.1 ± 40.3	14.1 ± 23.4	22.7 ± 41.3	32.1 ± 4.9	31.5 ± 5.1	32.2 ± 4.8
Median (IQR)	7.9 (0–26.9)	7.9 (0–16.8)	7.9 (0–26.9)	31 (28.9–34.2)	30.8 (28.1–33.2)	31.0 (29.0–34.5)
Waist circumference, cm						
Mean ± SD	106 ± 14.2	108 ± 13.3	103 ± 14.3	98.0 ± 14.1	105 ± 16.1	96.6 ± 13.2
Median (IQR)	104 (96.2–113)	107 (98.3–114)	104 (96.1–113)	97.0 (89.5–106)	103 (93.3–113)	96.5 (89.0–105)
Alcohol intake, g						
Mean ± SD	2.2 ± 5.7	3.5 ± 9.6	2.2 ± 5.3	6.3 ± 21.5	1.1 ± 3.6	7.3 ± 23.3
Median (IQR)	0 (0.0–1.0)	0 (0.0–1.0)	0 (0.0–0.7)	0.1 (0.0–2.9)	0 (0.0–0.4)	0.2 (0.01–4.0)
BMI, kg/m^2^						
Mean ± SD	33.3 ± 6.2	33.9 ± 5.9	33.2 ± 6.3	30.9 ± 6.4	33.8 ± 7.7	30.3 ± 6.0
Median (IQR)	31.7 (28.8–36.3)	32.9 (29.3–37.2)	31.5 (28.8–36.3)	30.0 (26.7–33.9)	33.0 (28.3–36.8)	29.8 (26.4–33.6)
Use of antihypertensive medications, yes, n (%)	268 (28.9)	25 (36.2)	243 (28.3)	119 (40.5)	27 (56.3)	92 (37.4)
Use of statins or other lipid-lowering medications, yes, n (%)	82 (8.9)	7 (10.1)	75 (8.7)	75 (25.5)	18 (37.5)	57 (23.2)

Abbreviations used: SOALS: San Juan Overweight Adult Longitudinal Study; BPRHS: Boston Puerto Rican Health Study; SD: standard deviation; IQR: interquartile range; ^a^ Education in the BPRHS was categorized as: less than high school, high school, and some college or more. ^b^ Physical activity in BPRHS is a physical activity score.

**Table 2:** Multivariable-adjusted pooled fixed-effects Incidence Rate Ratios (95% confidence intervals) for progression to type 2 diabetes and/or prediabetes, according to 1 standard deviation change in predefined clusters of metabolites in the San Juan Overweight Adult Longitudinal Study (SOALS) and Boston Puerto Rican Health Study (BPRHS) cohorts.

Cluster Name	Number of Included Metabolites	Among All Participants (*n* = 1221)		Among Participants with Prediabetes at Baseline (*n* = 751)		Among Participants with Normal Blood Glucose Concentration at Baseline (*n* = 459)		Among All Participants (*n* = 1221)	
		Progression to Type 2 Diabetes		Progression to Diabetes		Progression to Prediabetes		Progression to Prediabetes or Type 2 Diabetes	
		Multivariable-Adjusted IRR (95% CI)	*p*-Value	Multivariable-Adjusted IRR (95% CI)	*p*-Value	Multivariable-Adjusted IRR (95% CI)	*p*-Value	Multivariable-Adjusted IRR (95% CI)	*p*-Value
Sphingolipids ^1^	18	0.94 (0.67; 1.31)	0.72	0.86 (0.60; 1.23)	0.41	0.82 (0.63; 1.08)	0.16	0.85 (0.69; 1.04)	0.12
BCAA metabolism ^2^	15	1.39 (0.92; 2.09)	0.11	1.19 (0.76; 1.86)	0.44	1.22 (0.88; 1.70)	0.23	1.15 (0.89; 1.48)	0.28
BCAA and aromatic amino acid metabolism ^3^	13	1.87 (1.28; 2.73)	0.001	1.62 (1.09; 2.41)	0.02	1.13 (0.84; 1.54)	0.42	1.20 (0.95; 1.51)	0.13
Acyl cholines ^4^	9	0.77 (0.55; 1.07)	0.12	0.79 (0.55; 1.13)	0.19	0.81 (0.62; 1.07)	0.14	0.88 (0.71; 1.08)	0.23
Aromatic amino acid metabolism ^5^	11	0.96 (0.68; 1.33)	0.79	0.88 (0.62; 1.26)	0.50	1.01 (0.77; 1.34)	0.92	0.99 (0.81; 1.23)	0.96
Cell membrane components ^6^	8	1.54 (1.04; 2.27)	0.03	1.31 (0.87; 1.98)	0.20	1.09 (0.78; 1.52)	0.62	1.24 (0.97; 1.58)	0.08
Glucose transport ^7^	6	1.27 (0.88; 1.83)	0.20	1.13 (0.77; 1.66)	0.53	0.79 (0.57; 1.07)	0.13	0.96 (0.76; 1.21)	0.72
Fatty acid biosynthesis ^8^	6	1.16 (0.91; 1.48)	0.22	1.11 (0.86; 1.44)	0.43	1.06 (0.87; 1.29)	0.54	1.02 (0.88; 1.19)	0.77
Sugar metabolism ^9^	5	1.32 (0.90; 1.94)	0.15	1.19 (0.80; 1.77)	0.38	0.93 (0.66; 1.30)	0.67	1.01 (0.79; 1.29)	0.91

Multivariable-adjusted Poisson regression models included the following confounders: age, sex, smoking, education, family history of diabetes (SOALS only), METs of physical activity (physical activity score in the BPRHS), waist circumference (cm), BMI (kg/m^2^), alcohol consumption (g/d), use of antihypertensive medication, statins or other lipid lowering medication. ^1^ Cluster included: sphingomyelin (d18:2/21:0, d16:2/23:0)*, sphingomyelin (d18:2/23:0, d18:1/23:1, d17:1/24:1)*, sphingomyelin (d18:2/23:1)*, sphingomyelin (d18:2/16:0, d18:1/16:1)*, sphingomyelin (d18:2/14:0, d18:1/14:1)*, sphingomyelin (d18:1/22:1, d18:2/22:0, d16:1/24:1)*, sphingomyelin (d17:2/16:0, d18:2/15:0)*, sphingomyelin (d18:1/19:0, d19:1/18:0)*, sphingomyelin (d18:1/22:2, d18:2/22:1, d16:1/24:2)*, sphingomyelin (d18:1/18:1, d18:2/18:0), 5alpha-androstan-3alpha,17beta-diol disulfate, sphingomyelin (d17:1/16:0, d18:1/15:0, d16:1/17:0)*, glycosyl-N-behenoyl-sphingadienine (d18:2/22:0)*, pyroglutamine*, creatinine, guanidinoacetate, 5-oxoproline, N6-carbamoylthreonyladenosine. ^2^ Cluster included: formiminoglutamate, 3-hydroxyisobutyrate, hydantoin-5-propionic acid, kynurenate, urea, picolinate, N-acetylkynurenine (2), leucine, 2-aminoadipate, glycinel, N-acetylglucosaminylasparagine, 6-oxopiperidine-2-carboxylate, N6-acetyllysine, gamma-glutamylglycine, 3-methylcytidine. ^3^ Cluster included: 2-hydroxy-3-methylvalerate, indolelactate, phenyllactate (PLA), alpha-hydroxyisocaproate, N6,N6,N6-trimethyllysine, 3-methyl-2-oxovalerate, phenylpyruvate, imidazole lactate, beta-hydroxyisovalerate, 4-methoxyphenol sulfate, 4-methyl-2-oxopentanoate, alpha-hydroxyisovalerate, 3-methyl-2-oxobutyrate. ^4^ Cluster included: 1-oleoyl-2-docosahexaenoyl-GPC (18:1/22:6)*, docosahexaenoylcholine, arachidonoylcholine, stearoylcholine*, eicosenoylcarnitine (C20:1)*, palmitoylcholine, 3-(4-hydroxyphenyl)lactate, 1-palmitoleoyl-2-linolenoyl-GPC (16:1/18:3)*, dihomo-linoleoylcarnitine (C20:2)*. ^5^ Cluster included: phenylacetylglutamine, isovalerylglycine, isobutyrylglycine, indole-3-carboxylic acid, p-cresol-glucuronide*, phenylacetylglutamate, phenylacetate, 3-methylglutaconate, 5alpha-androstan-3beta,17alpha-diol disulfate, L-urobilin, 3-hydroxy-3-methylglutarate. ^6^ Cluster included: 1-palmitoyl-2-palmitoleoyl-GPC (16:0/16:1)*, N-acetylputrescine, 1-palmitoleoyl-GPC (16:1)*, 2-palmitoleoyl-GPC (16:1)*, sphingomyelin (d18:1/20:1, d18:2/20:0)*, N1-methyladenosine, 7-methylguanine, sphingomyelin (d18:2/24:2)*. ^7^ Cluster included: 1,2-dipalmitoyl-GPC (16:0/16:0), 1,5-anhydroglucitol (1,5-AG), 2-linoleoylglycerol (18:2), N-acetyltaurine, glycosyl-N-palmitoyl-sphingosine (d18:1/16:0), linoleoylcholine*. ^8^ Cluster included: eicosenoate (20:1), 3-hydroxybutyrate (BHBA), 3-hydroxydecanoate, docosadienoate (22:2n6), 2-hydroxybutyrate/2-hydroxyisobutyrate, oleate/vaccenate (18:1). ^9^ Cluster included: maltose, glycerol 3-phosphate, mannitol/sorbitol, beta-citrylglutamate, phosphate.

## Data Availability

Data are available upon reasonable request, following approval of the analysis plan from the study investigators. For more information on requesting data from the BPRHS (https://www.uml.edu/Research/UML-CPH/Research/bprhs/, accessed on 5 May 2022) and SOALS (http://soals.rcm.upr.edu/, accessed on 5 May 2022) please refer to their respective websites.

## Supplementary Materials

The following supporting information can be downloaded at: https://www.mdpi.com/article/10.3390/metabo12060513/s1, Table S1: Multivariable-adjusted pooled random-effects Incidence Rate Ratios (95% confidence intervals) for progression to type 2 diabetes and/or prediabetes, according to 1 standard deviation change in predefined clusters of metabolites in the San Juan Overweight Adult Longitudinal Study (SOALS) and Boston Puerto Rican Health Study (BPRHS) cohorts. Table S2. Multivariable-adjusted Incidence Rate Ratios (95% confidence intervals) for progression to type 2 diabetes, according to 1 standard deviation change in predefined clusters of metabolites in the San Juan Overweight Adult Longitudinal Study (SOALS) and Boston Puerto Rican Health Study (BPRHS) cohorts. Table S3. Incidence Rate Ratios (95% confidence intervals) for progression to type 2 diabetes, according to 1 standard deviation change in each metabolite in the San Juan Overweight Adult Longitudinal Study (SOALS). Table S4. Incidence Rate Ratios (95% confidence intervals) for progression to type 2 diabetes, according to 1 standard deviation change in each metabolite in the Boston Puerto Rican Health Study (BPRHS). Table S5. Incidence Rate Ratios (95% confidence intervals) for progression from prediabetes to type 2 diabetes, according to 1 standard deviation change in each metabolite, for select metabolites in the San Juan Overweight Adult Longitudinal Study (SOALS) and Boston Puerto Rican Health Study (BPRHS) cohorts.
