# Supplementary material for: Novel Plasma Metabolomic Markers Associated with Diabetes Progression in Older Puerto Ricans
Source: Metabolites. 2022 Jun 2;12(6):513. doi: 10.3390/metabo12060513 (PMC9227184; doi:10.3390/metabo12060513)
Supplement: Supplementary file 1 [file metabolites-12-00513-s001.zip › metabolites-1737887-supplementary.pdf]

SUPPLEMENTAL MATERIAL

Supplemental Table S1. Multivariable-adjusted pooled random-effects Incidence Rate Ratios (95% confidence intervals) for progression to type 2 diabetes and/or prediabetes, according to 1 standard deviation change in predefined clusters of metabolites in the San Juan Overweight Adult Longitudinal Study (SOALS) and Boston Puerto Rican Health Study (BPRHS) cohorts.

Cluster name	Number of included metabolites	Among all participants (n=1,221)		Among participants with prediabetes at baseline (n=751)		Among participants with normal blood glucose concentration at baseline (n=459)		Among all participants (n=1,221)	
		Progression to type 2 diabetes		Progression to type 2 diabetes		Progression to prediabetes		Progression to prediabetes or type 2 diabetes	
		**Multivariable-adjusted IRR (95% CI)**	**p-value**	**Multivariable-adjusted IRR (95% CI)**	**p-value**	**Multivariable-adjusted IRR (95% CI)**	**p-value**	**Multivariable-adjusted IRR (95% CI)**	**p-value**
**Sphingolipids[1]**	18	0.98 (0.60; 1.60)	0.93	0.86 (0.60; 1.23)	0.41	1.01 (0.48; 2.14)	0.97	0.85 (0.68; 1.07)	0.16
**BCAA Metabolism[2]**	15	1.39 (0.92; 2.09)	0.11	1.19 (0.76; 1.86)	0.44	1.30 (0.80; 2.10)	0.29	1.15 (0.89; 1.48)	0.28
**BCAA and aromatic amino acid metabolism[3]**	13	1.87 (1.28; 2.73)	0.001	1.62 (1.09; 2.41)	0.02	1.19 (0.77; 1.82)	0.44	1.22 (0.91; 1.5)	0.19
**Acyl cholines[4]**	9	0.77 (0.55; 1.07)	0.12	0.79 (0.55; 1.13)	0.19	0.95 (0.49; 1.84)	0.88	0.88 (0.71; 1.08)	0.23
**Aromatic amino acid metabolism[5]**	11	0.96 (0.68; 1.33)	0.79	0.88 (0.62; 1.26)	0.50	1.14 (0.64; 2.01)	0.66	1.03 (0.75; 1.41)	0.85
**Cell membrane components[6]**	8	1.54 (1.04; 2.27)	0.03	1.31 (0.87; 1.98)	0.20	1.12 (0.74; 1.71)	0.59	1.26 (0.95; 1.67)	0.11
**Glucose transport[7]**	6	1.27 (0.88; 1.83)	0.20	1.13 (0.77; 1.66)	0.53	1.03 (0.40; 2.66)	0.95	1.10 (0.60; 2.00)	0.76
**Fatty Acid Biosynthesis[8]**	6	1.16 (0.91; 1.48)	0.22	1.11 (0.86; 1.44)	0.43	1.06 (0.87; 1.29)	0.54	1.02 (0.88; 1.19)	0.77
**Sugar Metabolism[9]**	5	1.32 (0.90; 1.94)	0.15	1.19 (0.80; 1.77)	0.38	0.93 (0.66; 1.30)	0.67	1.01 (0.79; 1.29)	0.91

Multivariable-adjusted Poisson regression models included the following confounders: age, sex, smoking, education, family history of diabetes (SOALS only), METs of physical activity (physical activity score in the BPRHS), waist circumference (cm), BMI (kg/m2), alcohol consumption (g/day), use of antihypertensive medications and statins or other lipid lowering medications. [1]Cluster included: sphingomyelin (d18:2/21:0, d16:2/23:0)*, sphingomyelin (d18:2/23:0, d18:1/23:1, d17:1/24:1)*, sphingomyelin (d18:2/23:1)*, sphingomyelin (d18:2/16:0, d18:1/16:1)*, sphingomyelin (d18:2/14:0, d18:1/14:1)*, sphingomyelin (d18:1/22:1, d18:2/22:0, d16:1/24:1)*, sphingomyelin (d17:2/16:0, d18:2/15:0)*, sphingomyelin (d18:1/19:0, d19:1/18:0)*, sphingomyelin (d18:1/22:2, d18:2/22:1, d16:1/24:2)*, sphingomyelin (d18:1/18:1, d18:2/18:0), 5alpha-androstan-3alpha,17beta-diol disulfate, sphingomyelin (d17:1/16:0, d18:1/15:0, d16:1/17:0)*, glycosyl-N-behenoyl-sphingadienine (d18:2/22:0)*, pyroglutamine*, creatinine, guanidinoacetate, 5-oxoproline, N6-carbamoylthreonyladenosine. [2]Cluster included: formiminoglutamate, 3-hydroxy-isobutyrate, hydantoin-5-propionic acid, kynurenate, urea, picolinate, N-acetylkynurenine (2), leucine, 2-aminoadipate, glycinel, N-acetylglucosaminylasparagine, 6-oxopiperidine-2-carboxylate, N6-acetyllysine, gamma-glutamylglycine, 3-methylcytidine. [3]Cluster included: 2-hydroxy-3-methylvalerate, indolelactate,

phenyllactate (PLA), alpha-hydroxyisocaproate, N6,N6,N6-trimethyllysine, 3-methyl-2-oxovalerate, phenylpyruvate, imidazole lactate, beta-hydroxyisovalerate, 4-methoxyphenol sulfate, 4-methyl-2-oxopentanoate, alpha-hydroxyisovalerate, 3-methyl-2-oxobutyrate. [4]Cluster included: 1-oleoyl-2-docosahexaenoyl-GPC (18:1/22:6)*, docosahexaenoylcholine, arachidonoylcholine, stearoylcholine*, eicosenoylcarnitine (C20:1)*, palmitoylcholine, 3-(4-hydroxyphenyl)lactate, 1-palmitoleoyl-2-linolenoyl-GPC (16:1/18:3)*, dihomo-linoleoylcarnitine (C20:2)*. [5]Cluster included: phenylacetylglutamine, isovalerylglycine, isobutyrylglycine, indole-3-carboxylic acid, p-cresol-glucuronide*, phenylacetylglutamate, phenylacetate, 3-methylglutaconate, 5alpha-androstan-3beta,17alpha-diol disulfate, L-urobilin, 3-hydroxy-3-methylglutarate. [6]Cluster included: 1-palmitoyl-2-palmitoleoyl-GPC (16:0/16:1)*, N-acetylputrescine, 1-palmitoleoyl-GPC (16:1)*, 2-palmitoleoyl-GPC (16:1)*, sphingomyelin (d18:1/20:1, d18:2/20:0)*, N1-methyladenosine, 7-methylguanine, sphingomyelin (d18:2/24:2)*. [7]Cluster included: 1,2-dipalmitoyl-GPC (16:0/16:0), 1,5-anhydroglucitol (1,5-AG), 2-linoleoylglycerol (18:2), N-acetyltaurine, glycosyl-N-palmitoyl-sphingosine (d18:1/16:0), linoleoylcholine*. [8]Cluster included: eicosenoate (20:1), 3-hydroxybutyrate (BHBA), 3-hydroxydecanoate, docosadienoate (22:2n6), 2-hydroxybutyrate/2-hydroxyisobutyrate, oleate/vaccenate (18:1). [9]Cluster included: maltose, glycerol 3-phosphate, mannitol/sorbitol, beta-citrylglutamate, phosphate.

Supplemental Table S2. Multivariable-adjusted Incidence Rate Ratios (95% confidence intervals) for progression to type 2 diabetes, according to 1 standard deviation change in predefined clusters of metabolites in the San Juan Overweight Adult Longitudinal Study (SOALS) and Boston Puerto Rican Health Study (BPRHS) cohorts.

**SOALS**									
**Cluster name**	**Number of included metabolites**	**Among all participants (n=927)**		**Among those with prediabetes at baseline (n=528)**		**Among those with normal glycemic levels at baseline (n=391)**		**Among all participants (n=927)**	
		Progression to type 2 diabetes		Progression to type 2 diabetes		Progression to prediabetes		Progression to prediabetes or type 2 diabetes	
		**Multivariable-adjusted IRR (95% CI)**	**p-value**	**Multivariable-adjusted IRR (95% CI)**	**p-value**	**Multivariable-adjusted IRR (95% CI)**	**p-value**	**Multivariable-adjusted IRR (95% CI)**	**p-value**
Sphingolipids[1]	18	0.79 (0.52;1.19)	0.25	0.76 (0.48; 1.21)	0.25	0.76 (0.57; 1.02)	0.06	0.80 (0.63; 1.01)	0.06
BCAA Metabolism[2]	15	1.63 (0.95; 2.78)	0.07	1.39 (0.79; 2.47)	0.26	1.10 (0.76; 1.59)	0.60	1.10 (0.81; 1.48)	0.54
BCAA and aromatic amino acid metabolism[3]	13	2.12 (1.29; 3.49)	0.003	1.93 (1.12; 3.32)	0.02	1.07 (0.77; 1.47)	0.70	1.09 (0.83; 1.44)	0.52
Acyl cholines[4]	9	0.81 (0.54; 1.24)	0.33	0.84 (0.53; 1.33)	0.45	0.72 (0.53; 0.98)	0.04	0.84 (0.66; 1.07)	0.17
Aromatic amino acid metabolism[5]	11	0.90 (0.59; 1.38)	0.64	0.82 (0.52; 1.29)	0.39	0.90 (0.66; 1.23)	0.50	0.91 (0.71; 1.16)	0.45

Cell membrane components[6]	8	1.53 (0.94; 2.51)	0.09	1.40 (0.84; 2.34)	0.20	1.01 (0.71; 1.45)	0.95	1.15 (0.87; 1.52)	0.33
Glucose transport[7]	6	1.26 (0.81; 1.97)	0.30	1.14 (0.72; 1.82)	0.58	0.68 (0.48; 0.95)	0.03	0.83 (0.64; 1.08)	0.17
Fatty Acid Biosynthesis[8]	6	1.25 (0.92; 1.71)	0.16	1.15 (0.82; 1.61)	0.42	1.07 (0.86; 1.32)	0.55	1.06 (0.89; 1.27)	0.49
Sugar Metabolism[9]	5	1.40 (0.85; 2.33)	0.19	1.26 (0.74; 2.15)	0.40	0.94 (0.65; 1.36)	0.75	0.95 (0.71; 1.27)	0.71

(continued)

**BPRHS**									
**Cluster name**	**Number of included metabolites**	**Among all participants (n=294)**		**Among those with prediabetes at baseline (n=223)**		**Among those with normal glycemic levels at baseline (n=68)**		**Among all participants (n=294)**	
		Progression to diabetes		Progression to diabetes		Progression to prediabetes		Progression to prediabetes or diabetes	
		**Multivariable-adjusted IRR (95% CI)**	**p-value**	**Multivariable-adjusted IRR (95% CI)**	**p-value**	**Multivariable-adjusted IRR (95% CI)**	**p-value**	**Multivariable-adjusted IRR (95% CI)**	**p-value**
Sphingolipids[1]	18	1.31 (0.75; 2.29)	0.35	1.04 (0.57; 1.87)	0.90	1.69 (0.70; 4.05)	0.24	1.04 (0.67; 1.60)	0.87
BCAA Metabolism[2]	15	1.12 (0.59; 2.10)	0.73	0.94 (0.46; 1.91)	0.85	1.87 (0.89; 3.92)	0.10	1.29 (0.81; 2.05)	0.29
BCAA and aromatic amino acid metabolism[3]	13	1.57 (0.87; 2.81)	0.13	1.32 (0.73; 2.37)	0.36	1.86 (0.74; 4.68)	0.19	1.51 (0.97; 2.33)	0.07
Acyl cholines[4]	9	0.70 (0.41; 1.20)	0.19	0.70 (0.39; 1.25)	0.23	1.43 (0.74; 2.79)	0.29	0.99 (0.65; 1.50)	0.97
Aromatic amino acid metabolism[5]	11	1.04 (0.61; 1.77)	0.88	1.00 (0.56; 1.78)	1.00	1.63 (0.88; 3.02)	0.12	1.27 (0.85; 1.90)	0.25
Cell membrane components[6]	8	1.55 (0.82; 2.92)	0.18	1.17 (0.59; 2.31)	0.66	1.74 (0.71; 4.26)	0.23	1.58 (0.96; 2.61)	0.07

Glucose transport[7]	6	1.28 (0.68; 2.42)	0.44	1.11 (0.57; 2.16)	0.76	1.81 (0.80; 4.06)	0.15	1.55 (0.95; 2.51)	0.08
Fatty Acid Biosynthesis[8]	6	1.04 (0.71; 1.54)	0.84	1.06 (0.71; 1.58)	0.78	1.04 (0.64; 1.69)	0.89	0.92 (0.69; 1.23)	0.57
Sugar Metabolism[9]	5	1.22 (0.68; 2.18)	0.51	1.12 (0.62; 2.02)	0.70	0.87 (0.39; 1.96)	0.73	1.20 (0.76; 1.87)	0.43

Multivariable Poisson regression models included the following confounders: age, sex, smoking, education, family history of diabetes (SOALS only), METs of physical activity (physical activity score in the BPRHS), waist circumference (cm), BMI (kg/m^2), alcohol consumption (g/day), use of antihypertensive medications and statins or other lipid lowering medications. [1]Cluster included: sphingomyelin (d18:2/21:0, d16:2/23:0)*, sphingomyelin (d18:2/23:0, d18:1/23:1, d17:1/24:1)*, sphingomyelin (d18:2/23:1)*, sphingomyelin (d18:2/16:0, d18:1/16:1)*, sphingomyelin (d18:2/14:0, d18:1/14:1)*, sphingomyelin (d18:1/22:1, d18:2/22:0, d16:1/24:1)*, sphingomyelin (d17:2/16:0, d18:2/15:0)*, sphingomyelin (d18:1/19:0, d19:1/18:0)*, sphingomyelin (d18:1/22:2, d18:2/22:1, d16:1/24:2)*, sphingomyelin (d18:1/18:1, d18:2/18:0), 5alpha-androstan-3alpha,17beta-diol disulfate, sphingomyelin (d17:1/16:0, d18:1/15:0, d16:1/17:0)*, glycosyl-N-behenoyl-sphingadienine (d18:2/22:0)*, pyroglutamine*, creatinine, guanidinoacetate, 5-oxoproline, N6-carbamoylthreonyladenosine. [2]Cluster included: formiminoglutamate, 3-hydroxyisobutyrate, hydantoin-5-propionic acid, kynurenate, urea, picolinate, N-acetylkynurenine (2), leucine, 2-aminoadipate, glycinel, N-acetylglucosaminylasparagine, 6-oxopiperidine-2-carboxylate, N6-acetyllysine, gamma-glutamylglycine, 3-methylcytidine. [3]Cluster included: 2-hydroxy-3-methylvalerate, indolelactate, phenyllactate (PLA), alpha-hydroxyisocaproate, N6,N6,N6-trimethyllysine, 3-methyl-2-oxovalerate, phenylpyruvate, imidazole lactate, beta-hydroxyisovalerate, 4-methoxyphenol sulfate, 4-methyl-2-oxopentanoate, alpha-hydroxyisovalerate, 3-methyl-2-oxobutyrate. [4]Cluster included: 1-oleoyl-2-docosahexaenoyl-GPC (18:1/22:6)*, docosahexaenoylcholine, arachidonoylcholine, stearoylcholine*, eicosenoylcarnitine (C20:1)*, palmitoylcholine, 3-(4-hydroxyphenyl)lactate, 1-palmitoleoyl-2-linolenoyl-GPC (16:1/18:3)*, dihomo-linoleoylcarnitine (C20:2)*. [5]Cluster included: phenylacetylglutamine, isovalerylglycine, isobutyrylglycine, indole-3-carboxylic acid, p-cresol-glucuronide*, phenylacetylglutamate, phenylacetate, 3-methylglutaconate, 5alpha-androstan-3beta,17alpha-diol disulfate, L-urobilin, 3-hydroxy-3-methylglutarate. [6]Cluster included: 1-palmitoyl-2-palmitoleoyl-GPC (16:0/16:1)*, N-acetylputrescine, 1-palmitoleoyl-GPC (16:1)*, 2-palmitoleoyl-GPC (16:1)*, sphingomyelin (d18:1/20:1, d18:2/20:0)*, N1-methyladenosine, 7-methylguanine, sphingomyelin (d18:2/24:2)*. [7]Cluster included: 1,2-dipalmitoyl-GPC (16:0/16:0), 1,5-anhydroglucitol (1,5-AG), 2-linoleoylglycerol (18:2), N-acetyltaurine, glycosyl-N-palmitoyl-sphingosine (d18:1/16:0), linoleoylcholine*. [8]Cluster included: eicosenoate (20:1), 3-hydroxybutyrate (BHBA), 3-hydroxydecanoate, docosadienoate (22:2n6), 2-hydroxybutyrate/2-hydroxyisobutyrate, oleate/vaccenate (18:1). [9]Cluster included: maltose, glycerol 3-phosphate, mannitol/sorbitol, beta-citrylglutamate, phosphate.

Supplemental Table S3. Incidence Rate Ratios (95% confidence intervals) for progression to type 2 diabetes, according to 1 standard deviation change in each metabolite in the San Juan Overweight Adult Longitudinal Study (SOALS).

Metabolite	Model 1 IRR (95% CI)	FDR p-value	Model 2 IRR (95% CI)	FDR p-value	Model 3 IRR (95% CI)	FDR p-value	SUPER-PATHWAY	SUB-PATHWAY
3-methyl-2-oxovalerate	1.77 (1.32; 2.39)	0.006	1.90 (1.4; 2.57)	0.005	1.87 (1.38; 2.54)	0.006	Amino Acid	Leucine, Isoleucine and Valine Metabolism
3-methyl-2-oxobutyrate	1.68 (1.28; 2.20)	0.006	1.74 (1.31; 2.31)	0.006	1.74 (1.30; 2.31)	0.008	Amino Acid	Leucine, Isoleucine and Valine Metabolism

1-palmitoyl-2-palmitoleoyl-GPC (16:0/16:1)*	1.64 (1.29; 2.08)	0.006	1.59 (1.24; 2.03)	0.007	1.59 (1.24; 2.03)	0.009	Lipid	Phosphatidylcholine (PC)
4-methyl-2-oxopentanoate	1.61 (1.19; 2.18)	0.03	1.73 (1.27; 2.38)	0.02	1.72 (1.25; 2.36)	0.02	Amino Acid	Leucine, Isoleucine and Valine Metabolism
5-oxoproline	1.48 (1.16; 1.88)	0.03	1.51 (1.18; 1.94)	0.02	1.50 (1.17; 1.93)	0.02	Amino Acid	Glutathione Metabolism
2-hydroxy-3-methylvalerate	1.64 (1.23; 2.18)	0.02	1.63 (1.21; 2.19)	0.02	1.62 (1.2; 2.18)	0.02	Amino Acid	Leucine, Isoleucine and Valine Metabolism
isoleucine	1.47 (1.12; 1.91)	0.03	1.52 (1.16; 2.00)	0.03	1.52 (1.15; 2.00)	0.03	Amino Acid	Leucine, Isoleucine and Valine Metabolism
ornithine	1.43 (1.12; 1.81)	0.03	1.45 (1.13; 1.86)	0.03	1.47 (1.14; 1.90)	0.03	Amino Acid	Urea cycle; Arginine and Proline Metabolism
3-hydroxyisobutyrate	1.49 (1.14; 1.93)	0.03	1.51 (1.16; 1.97)	0.03	1.49 (1.14; 1.95)	0.03	Amino Acid	Leucine, Isoleucine and Valine Metabolism
sphingomyelin (d18:1/20:1, d18:2/20:0)*	0.66 (0.51; 0.86)	0.03	0.67 (0.51; 0.87)	0.03	0.66 (0.51; 0.87)	0.03	Lipid	Sphingolipid Metabolism
sphingomyelin (d18:2/24:2)*	0.68 (0.53; 0.88)	0.03	0.66 (0.51; 0.87)	0.03	0.67 (0.51; 0.87)	0.03	Lipid	Sphingolipid Metabolism
leucine	1.5 (1.14; 1.97)	0.03	1.57 (1.18; 2.09)	0.03	1.57 (1.18; 2.09)	0.03	Amino Acid	Leucine, Isoleucine and Valine Metabolism
formiminoglutamate	1.54 (1.17; 2.03)	0.03	1.52 (1.14; 2.02)	0.04	1.52 (1.14; 2.02)	0.03	Amino Acid	Histidine Metabolism
1-oleoyl-2-docosahexaenoyl-GPC (18:1/22:6)*	0.69 (0.54; 0.88)	0.03	0.69 (0.53; 0.9)	0.04	0.68 (0.52; 0.89)	0.03	Lipid	Phosphatidylcholine (PC)
valine	1.43 (1.1; 1.86)	0.05	1.48 (1.13; 1.94)	0.04	1.47 (1.12; 1.94)	0.04	Amino Acid	Leucine, Isoleucine and Valine Metabolism

tyrosine	1.42 (1.11; 1.81)	0.03	1.37 (1.07; 1.75)	0.06	1.38 (1.08; 1.77)	0.07	Amino Acid	Tyrosine metabolism
alpha-hydroxyisovalerate	1.47 (1.12; 1.92)	0.04	1.45 (1.09; 1.92)	0.06	1.44 (1.09; 1.91)	0.07	Amino Acid	Leucine, Isoleucine and Valine Metabolism
2-hydroxybutyrate/2-hydroxyisobutyrate	1.38 (1.07; 1.78)	0.07	1.43 (1.1; 1.86)	0.05	1.41 (1.08; 1.83)	0.07	Amino Acid	Glutathione Metabolism
3-(4-hydroxyphenyl) lactate (HPLA)	1.4 (1.06; 1.84)	0.08	1.45 (1.09; 1.94)	0.06	1.44 (1.07; 1.92)	0.08	Amino Acid	Tyrosine Metabolism
2-aminoadipate	1.42 (1.09; 1.84)	0.05	1.42 (1.08; 1.86)	0.07	1.4 (1.06; 1.84)	0.09	Amino Acid	Lysine Metabolism
alpha-hydroxyisocaproate	1.41 (1.04; 1.91)	0.12	1.48 (1.08; 2.03)	0.08	1.47 (1.07; 2.02)	0.09	Amino Acid	Leucine, Isoleucine and Valine Metabolism
imidazole lactate	0.76 (0.59; 0.98)	0.14	0.75 (0.58; 0.98)	0.14	0.74 (0.57; 0.97)	0.13	Amino Acid	Histidine Metabolism
docosadienoate (22:2n6)	1.36 (1.07; 1.73)	0.06	1.33 (1.04; 1.7)	0.10	1.32 (1.03; 1.68)	0.13	Lipid	Polyunsaturated Fatty Acid (n3 and n6)
isovalerylglycine	0.76 (0.59; 0.96)	0.10	0.76 (0.59; 0.98)	0.14	0.76 (0.59; 0.98)	0.14	Amino Acid	Leucine, Isoleucine and Valine Metabolism
glycine	0.74 (0.58; 0.95)	0.08	0.76 (0.59; 0.98)	0.14	0.76 (0.59; 0.98)	0.14	Amino Acid	Glycine, Serine and Threonine Metabolism
2-palmitoleoyl-GPC* (16:1)*	1.29 (1.02; 1.64)	0.14	1.29 (1.02; 1.64)	0.14	1.29 (1.01; 1.64)	0.15	Lipid	Lysophospholipid
sphingomyelin (d18:1/22:2, d18:2/22:1, d16:1/24:2)*	0.77 (0.6; 0.99)	0.17	0.75 (0.58; 0.98)	0.14	0.76 (0.58; 0.99)	0.16	Lipid	Sphingolipid Metabolism
N6,N6,N6-trimethyllysine	1.25 (0.97; 1.62)	0.26	1.3 (1; 1.69)	0.18	1.29 (1.00; 1.68)	0.20	Amino Acid	Lysine Metabolism
3-methylglutaconate	1.24 (0.97; 1.6)	0.26	1.32 (1.01; 1.72)	0.15	1.30 (0.99; 1.70)	0.20	Amino Acid	Leucine, Isoleucine and Valine Metabolism
betaine	0.81 (0.63; 1.04)	0.28	0.79 (0.61; 1.02)	0.24	0.79 (0.61; 1.02)	0.25	Amino Acid	Glycine, Serine and Threonine Metabolism

indolelactate	1.25 (0.95; 1.65)	0.28	1.29 (0.97; 1.71)	0.24	1.28 (0.96; 1.69)	0.26	Amino Acid	Tryptophan Metabolism
2-linoleoylglycerol (18:2)	1.27 (0.99; 1.62)	0.21	1.25 (0.98; 1.6)	0.24	1.25 (0.97; 1.60)	0.26	Lipid	Monoacylglycerol
trimethylamine N-oxide	1.23 (0.97; 1.57)	0.26	1.25 (0.98; 1.6)	0.24	1.24 (0.97; 1.59)	0.26	Lipid	Phospholipid Metabolism
hydantoin-5-propionate	1.23 (0.96; 1.58)	0.27	1.28 (0.98; 1.67)	0.23	1.26 (0.97; 1.64)	0.26	Amino Acid	Histidine Metabolism
sphingomyelin (d18:1/22:1, d18:2/22:0, d16:1/24:1)*	0.79 (0.62; 1.02)	0.24	0.79 (0.61; 1.03)	0.24	0.80 (0.61; 1.03)	0.26	Lipid	Sphingolipid Metabolism
sphingomyelin (d18:2/23:0, d18:1/23:1, d17:1/24:1)*	0.77 (0.59; 1.01)	0.22	0.78 (0.59; 1.03)	0.24	0.78 (0.59; 1.04)	0.27	Lipid	Sphingolipid Metabolism
sphingomyelin (d18:2/23:1)*	0.81 (0.62; 1.05)	0.28	0.79 (0.60; 1.04)	0.24	0.79 (0.60; 1.04)	0.28	Lipid	Sphingolipid Metabolism
sphingomyelin (d18:2/21:0, d16:2/23:0)*	0.78 (0.59; 1.04)	0.26	0.78 (0.57; 1.05)	0.28	0.78 (0.58; 1.05)	0.29	Lipid	Sphingolipid Metabolism
oleate/vaccenate (18:1)	1.26 (0.99; 1.6)	0.22	1.22 (0.95; 1.56)	0.31	1.20 (0.94; 1.54)	0.37	Lipid	Long Chain Fatty Acid
docosahexaenoylcholine	0.82 (0.64; 1.04)	0.28	0.84 (0.66; 1.07)	0.38	0.83 (0.66; 1.06)	0.37	Lipid	Fatty Acid Metabolism (Acyl Choline)
1,2-dipalmitoyl-GPC (16:0/16:0)	1.19 (0.93; 1.51)	0.40	1.19 (0.93; 1.52)	0.42	1.20 (0.93; 1.54)	0.38	Lipid	Phosphatidylcholine (PC)
arachidonoylcholine	0.84 (0.66; 1.07)	0.38	0.84 (0.66; 1.07)	0.38	0.84 (0.66; 1.07)	0.38	Lipid	Fatty Acid Metabolism (Acyl Choline)
eicosenoate (20:1n9 or 1n11)	1.23 (0.97; 1.57)	0.26	1.2 (0.94; 1.54)	0.37	1.19 (0.93; 1.52)	0.41	Lipid	Long Chain Fatty Acid
1-palmitoleoyl-GPC* (16:1)*	1.16 (0.92; 1.48)	0.46	1.19 (0.93; 1.52)	0.38	1.19 (0.93; 1.51)	0.41	Lipid	Lysophospholipid
N6-acetyllysine	0.87 (0.68; 1.11)	0.49	0.85 (0.66; 1.1)	0.49	0.85 (0.66; 1.09)	0.45	Amino Acid	Lysine Metabolism
N-acetylglucosaminylasparagine	0.89 (0.7; 1.13)	0.54	0.86 (0.67; 1.1)	0.50	0.86 (0.67; 1.10)	0.50	Carbohydrate	Aminosugar Metabolism
gamma-glutamylglycine	0.83 (0.65; 1.07)	0.38	0.85 (0.65; 1.1)	0.49	0.85 (0.65; 1.11)	0.50	Peptide	Gamma-glutamyl Amino Acid
glycerol 3-phosphate	1.14 (0.9; 1.44)	0.50	1.16 (0.91; 1.48)	0.49	1.16 (0.91; 1.48)	0.51	Lipid	Glycerolipid Metabolism

phenyllactate (PLA)	1.15 (0.87; 1.52)	0.55	1.17 (0.88; 1.55)	0.52	1.16 (0.87; 1.54)	0.54	Amino Acid	Phenylalanine Metabolism
5alpha-androstan-3alpha,17beta-diol disulfate	1.19 (0.87; 1.64)	0.50	1.18 (0.86; 1.62)	0.53	1.19 (0.87; 1.64)	0.54	Lipid	Androgenic Steroids
5alpha-androstan-3beta,17alpha-diol disulfate	0.86 (0.67; 1.1)	0.47	0.87 (0.67; 1.12)	0.52	0.87 (0.67; 1.12)	0.54	Lipid	Androgenic Steroids
N-acetylputrescine	1.16 (0.91; 1.47)	0.47	1.14 (0.89; 1.47)	0.52	1.14 (0.89; 1.46)	0.54	Amino Acid	Polyamine Metabolism
phosphate	1.14 (0.89; 1.45)	0.50	1.14 (0.89; 1.46)	0.52	1.14 (0.89; 1.45)	0.54	Energy	Oxidative Phosphorylation
1-palmitoleoyl-2-linolenoyl-GPC (16:1/18:3)*	1.15 (0.89; 1.48)	0.50	1.15 (0.88; 1.49)	0.52	1.15 (0.88; 1.49)	0.54	Lipid	Phosphatidylcholine (PC)
phenylpyruvate	1.18 (0.91; 1.53)	0.44	1.17 (0.9; 1.52)	0.51	1.15 (0.88; 1.50)	0.54	Amino Acid	Phenylalanine Metabolism
glycosyl-N-behenoyl-sphingadienine (d18:2/22:0)*	0.85 (0.67; 1.08)	0.44	0.87 (0.68; 1.1)	0.51	0.87 (0.68; 1.12)	0.54	Lipid	Ceramides
linoleoylcholine*	0.87 (0.68; 1.10)	0.49	0.88 (0.69; 1.12)	0.52	0.88 (0.69; 1.12)	0.54	Lipid	Fatty Acid Metabolism (Acyl Choline)
stearoylcholine*	0.87 (0.68; 1.11)	0.50	0.88 (0.69; 1.13)	0.53	0.88 (0.69; 1.13)	0.54	Lipid	Fatty Acid Metabolism (Acyl Choline)
sphingomyelin (d17:2/16:0, d18:2/15:0)*	0.87 (0.67; 1.14)	0.54	0.85 (0.64; 1.13)	0.52	0.85 (0.64; 1.14)	0.54	Lipid	Sphingolipid Metabolism
phenylalanine	1.15 (0.90; 1.48)	0.50	1.15 (0.88; 1.49)	0.52	1.15 (0.88; 1.50)	0.54	Amino Acid	Phenylalanine metabolism
carnitine	1.18 (0.93; 1.51)	0.40	1.15 (0.9; 1.48)	0.52	1.13 (0.88; 1.46)	0.55	Lipid	Carnitine metabolism
fumarate	0.87 (0.68; 1.12)	0.50	0.9 (0.7; 1.16)	0.62	0.89 (0.69; 1.14)	0.60	Energy	TCA Cycle
N6-carbamoylthreonyladenosine	1.18 (0.91; 1.53)	0.45	1.16 (0.88; 1.53)	0.52	1.14 (0.86; 1.50)	0.60	Nucleotide	Purine Metabolism, Adenine containing
palmitoylcholine	0.9 (0.71; 1.14)	0.60	0.89 (0.70; 1.14)	0.59	0.90 (0.70; 1.14)	0.62	Lipid	Fatty Acid Metabolism (Acyl Choline)
kynurenate	1.1 (0.85; 1.42)	0.68	1.14 (0.86; 1.49)	0.59	1.12 (0.85; 1.47)	0.62	Amino Acid	Tryptophan Metabolism

maltose	1.09 (0.86; 1.37)	0.68	1.11 (0.87; 1.42)	0.62	1.11 (0.87; 1.42)	0.62	Carbohydrate	Glycogen Metabolism
sphingomyelin (d18:1/18:1, d18:2/18:0)	0.9 (0.7; 1.17)	0.68	0.89 (0.68; 1.17)	0.62	0.89 (0.68; 1.16)	0.62	Lipid	Sphingolipid Metabolism
eicosenoylcarnitine (C20:1)*	1.09 (0.85; 1.40)	0.68	1.13 (0.87; 1.46)	0.59	1.12 (0.86; 1.44)	0.62	Lipid	Fatty Acid Metabolism (Acyl Carnitine)
sphingomyelin (d18:2/16:0, d18:1/16:1)*	0.92 (0.71; 1.19)	0.69	0.89 (0.69; 1.17)	0.62	0.90 (0.68; 1.17)	0.63	Lipid	Sphingolipid Metabolism
phenylacetylglutamate	0.91 (0.71; 1.15)	0.67	0.91 (0.71; 1.17)	0.68	0.90 (0.70; 1.16)	0.63	Peptide	Acetylated Peptides
N-acetylkynurenine (2)	1.1 (0.84; 1.44)	0.68	1.12 (0.85; 1.47)	0.62	1.11 (0.85; 1.47)	0.64	Amino Acid	Tryptophan Metabolism
1,5-anhydroglucitol (1,5-AG)	1.12 (0.87; 1.46)	0.60	1.1 (0.84; 1.45)	0.69	1.09 (0.83; 1.43)	0.75	Carbohydrate	Glycolysis, Gluconeogenesis, and Pyruvate Metabolism
citrulline	0.91 (0.71; 1.18)	0.68	0.92 (0.71; 1.2)	0.75	0.92 (0.71; 1.20)	0.75	Amino Acid	Urea Cycle; Arginine and Proline Metabolism
pyroglutamine*	1.10 (0.81; 1.50)	0.69	1.12 (0.82; 1.52)	0.69	1.11 (0.81; 1.50)	0.75	Amino Acid	Glutamate Metabolism
creatinine	0.89 (0.65; 1.21)	0.68	0.92 (0.67; 1.27)	0.81	0.90 (0.65; 1.25)	0.75	Amino Acid	Creatine Metabolism
picolinate	0.92 (0.71; 1.18)	0.69	0.94 (0.73; 1.22)	0.83	0.93 (0.71; 1.20)	0.76	Amino Acid	Tryptophan Metabolism
sphingomyelin (d18:2/14:0, d18:1/14:1)*	0.95 (0.72; 1.25)	0.83	0.9 (0.67; 1.22)	0.71	0.92 (0.68; 1.23)	0.76	Lipid	Sphingolipid Metabolism
dihomo-linoleoylcarnitine (C20:2)*	1.08 (0.85; 1.37)	0.70	1.09 (0.85; 1.40)	0.69	1.08 (0.84; 1.38)	0.76	Lipid	Fatty Acid Metabolism (Acyl Carnitine)
beta-hydroxyisovalerate	1.09 (0.84; 1.41)	0.69	1.09 (0.84; 1.42)	0.71	1.08 (0.83; 1.40)	0.77	Amino Acid	Leucine, Isoleucine and Valine Metabolism
phenylacetylglutamine	0.92 (0.73; 1.17)	0.69	0.94 (0.74; 1.20)	0.83	0.94 (0.74; 1.19)	0.77	Peptide	Acetylated Peptides

1-methyladenosine	1.07 (0.84; 1.37)	0.72	1.06 (0.82; 1.36)	0.83	1.07 (0.82; 1.38)	0.81	Nucleotide	Purine Metabolism, Adenine containing
indole-3-carboxylate	1.06 (0.83; 1.35)	0.80	1.06 (0.83; 1.36)	0.83	1.06 (0.83; 1.36)	0.81	Amino Acid	Tryptophan Metabolism
aspartate	1.09 (0.86; 1.39)	0.68	1.05 (0.82; 1.34)	0.86	1.05 (0.82; 1.34)	0.88	Amino Acid	Alanine and Aspartate Metabolism
Urea	0.94 (0.72; 1.21)	0.77	0.98 (0.75; 1.27)	0.94	0.95 (0.73; 1.24)	0.88	Amino Acid	Urea cycle; Arginine and Proline Metabolism
3-hydroxydecanoate	1.01 (0.8; 1.28)	0.96	1.04 (0.81; 1.33)	0.89	1.04 (0.82; 1.34)	0.88	Lipid	Fatty Acid, Monohydroxy
N-acetyltaurine	1.1 (0.85; 1.41)	0.68	1.06 (0.81; 1.38)	0.83	1.05 (0.80; 1.37)	0.88	Amino Acid	Methionine, Cysteine, SAM and Taurine Metabolism
beta-citrylglutamate	0.97 (0.77; 1.23)	0.91	0.96 (0.76; 1.23)	0.90	0.96 (0.75; 1.22)	0.88	Amino Acid	Glutamate Metabolism
mannitol/sorbitol	1.06 (0.83; 1.35)	0.80	1.06 (0.82; 1.36)	0.83	1.04 (0.81; 1.34)	0.90	Carbohydrate	Fructose, Mannose and Galactose Metabolism
sphingomyelin (d17:1/16:0, d18:1/15:0, d16:1/17:0)*	0.95 (0.74; 1.21)	0.81	0.95 (0.74; 1.23)	0.85	0.96 (0.75; 1.24)	0.90	Lipid	Sphingolipid Metabolism
choline	0.99 (0.77; 1.28)	0.96	0.97 (0.74; 1.27)	0.94	0.96 (0.73; 1.27)	0.90	Lipid	Phospholipid Metabolism
3-methylcytidine	1.06 (0.83; 1.36)	0.79	1.03 (0.79; 1.33)	0.94	1.04 (0.80; 1.34)	0.90	Nucleotide	Pyrimidine Metabolism, Cytidine containing
3-hydroxybutyrate (BHBA)	0.96 (0.75; 1.21)	0.83	0.97 (0.76; 1.23)	0.93	0.97 (0.76; 1.23)	0.90	Lipid	Ketone Bodies
6-oxopiperidine-2-carboxylate	1.03 (0.81; 1.31)	0.90	1.05 (0.82; 1.34)	0.85	1.03 (0.80; 1.31)	0.94	Amino Acid	Lysine Metabolism
7-methylguanine	1.02 (0.79; 1.32)	0.94	1.02 (0.79; 1.32)	0.97	1.02 (0.79; 1.33)	0.94	Nucleotide	Purine Metabolism, Guanine containing
3-hydroxy-3-methylglutarate	0.98 (0.76; 1.26)	0.94	1.00 (0.77; 1.29)	0.98	0.98 (0.75; 1.27)	0.94	Lipid	Mevalonate Metabolism

isobutyrylglycine (C4)	0.96 (0.75; 1.24)	0.87	1.03 (0.79; 1.33)	0.94	1.02 (0.79; 1.32)	0.95	Amino Acid	Leucine, Isoleucine and Valine Metabolism
L-urobilin	0.99 (0.75; 1.31)	0.96	0.98 (0.74; 1.3)	0.96	0.98 (0.74; 1.30)	0.95	Cofactors and Vitamins	Hemoglobin and Porphyrin Metabolism
glycosyl-N-palmitoyl-sphingosine (d18:1/16:0)	1.01 (0.80; 1.28)	0.96	1.00 (0.79; 1.27)	0.98	1.01 (0.80; 1.29)	0.96	Lipid	Ceramides
4-methoxyphenol sulfate	0.97 (0.76; 1.23)	0.90	0.99 (0.77; 1.26)	0.97	0.99 (0.77; 1.26)	0.97	Amino Acid	Tyrosine Metabolism
3-aminoisobutyrate	1.01 (0.79; 1.29)	0.96	1.01 (0.79; 1.29)	0.98	1.00 (0.78; 1.28)	0.99	Nucleotide	Pyrimidine Metabolism, Thymine containing
guanidinoacetate	0.99 (0.76; 1.28)	0.96	1.01 (0.77; 1.32)	0.98	1.00 (0.76; 1.31)	0.99	Amino Acid	Creatine Metabolism
p-cresol glucuronide*	0.99 (0.77; 1.25)	0.96	1.01 (0.79; 1.29)	0.98	1.00 (0.79; 1.28)	0.99	Amino Acid	Tyrosine Metabolism
sphingomyelin (d18:1/19:0, d19:1/18:0)*	0.99 (0.77; 1.28)	0.96	0.99 (0.76; 1.29)	0.98	1.00 (0.77; 1.30)	0.99	Lipid	Sphingolipid Metabolism
phenylacetate	1.00 (0.78; 1.27)	0.97	1.00 (0.79; 1.29)	0.98	1.00 (0.79; 1.28)	0.99	Amino Acid	Phenylalanine Metabolism

Model 1: Poisson regression models included the individual metabolites, age, and sex. Model 2: Model 1 + smoking, education, family history of diabetes, METs of physical activity (physical activity score in BPRHS), waist circumference (cm), BMI (kg/m^2), and alcohol consumption (g/day). Model 3: Model 2 + use of antihypertensive medications and statins or other lipid lowering medications. Benjamini-Hochberg FDR p-values were calculated for 104 metabolites.

Supplemental Table S4. Incidence Rate Ratios (95% confidence intervals) for progression to type 2 diabetes, according to 1 standard deviation change in each metabolite in the Boston Puerto Rican Health Study (BPRHS).

Metabolite	Model 1 IRR (95% CI)	FDR p-value	Model 2 IRR (95% CI)	FDR p-value	Model 3 IRR (95% CI)	FDR p-value	SUPER-PATHWAY	SUB-PATHWAY
3-methyl-2-oxovalerate	1.69 (1.25, 3.30)	0.05	1.77 (1.27; 2.46)	0.08	1.66 (1.19; 2.32)	0.31	Amino Acid	Leucine, Isoleucine and Valine Metabolism

3-methyl-2-oxobutyrate	1.60 (1.20; 2.13)	0.05	1.50 (1.11; 2.03)	0.22	1.44 (1.07; 1.95)	0.37	Amino Acid	Leucine, Isoleucine and Valine Metabolism
4-methyl-2-oxopentanoate	1.50 (1.10; 2.05)	0.23	1.60 (1.14; 2.26)	0.22	1.52 (1.08; 2.15)	0.37	Amino Acid	Leucine, Isoleucine and Valine Metabolism
isoleucine	1.65 (1.22; 2.22)	0.05	1.55 (1.12; 2.16)	0.22	1.50 (1.09; 2.08)	0.37	Amino Acid	Leucine, Isoleucine and Valine Metabolism
glycosyl-N-palmitoyl-sphingosine (d18:1/	1.21 (0.88; 1.67)	0.23	1.38 (0.98; 1.94)	0.84	1.55 (1.09; 2.19)	0.37	Lipid	Ceramides
3-aminoisobutyrate	0.79 (0.58; 1.08)	0.65	0.82 (0.59; 1.14)	0.94	0.89 (0.64; 1.23)	0.47	Nucleotide	Pyrimidine Metabolism, Thymine containing
1-palmitoyl-2-palmitoleoyl-GPC (16:0/16:1)*	1.20 (0.90; 1.61)	0.71	1.19 (0.87; 1.63)	0.94	1.14 (0.83; 1.58)	0.89	Lipid	Phosphatidylcholine (PC)
5-oxoproline	0.94 (0.70; 1.26)	0.84	0.90 (0.67; 1.22)	0.94	0.88 (0.64; 1.20)	0.89	Amino Acid	Glutathione Metabolism
2-hydroxy-3-methylvalerate	1.19 (0.88; 1.61)	0.71	1.19 (0.85; 1.68)	0.94	1.17 (0.83; 1.65)	0.89	Amino Acid	Leucine, Isoleucine and Valine Metabolism
ornithine	0.95 (0.69; 1.32)	0.87	0.73 (0.51; 1.04)	0.84	0.71 (0.50; 1.03)	0.89	Amino Acid	Urea cycle; Arginine and Proline Metabolism
3-hydroxyisobutyrate	1.43 (1.04; 1.95)	0.25	1.32 (0.93; 1.86)	0.94	1.31 (0.92; 1.85)	0.89	Amino Acid	Leucine, Isoleucine and Valine Metabolism
sphingomyelin (d18:1/20:1, d18:2/20:0)*	1.06 (0.79; 1.44)	0.84	1.07 (0.77; 1.48)	0.94	1.09 (0.78; 1.53)	0.89	Lipid	Sphingolipid Metabolism
sphingomyelin (d18:2/24:2)*	1.06 (0.77; 1.46)	0.84	1.03 (0.74; 1.44)	0.95	1.16 (0.83; 1.64)	0.89	Lipid	Sphingolipid Metabolism
leucine	1.65 (1.19; 2.30)	0.08	1.51 (1.05; 2.17)	0.55	1.43 (1.00; 2.05)	0.89	Amino Acid	Leucine, Isoleucine and Valine Metabolism

formiminoglutamate	1.23 (0.90; 1.68)	0.71	1.02 (0.72; 1.45)	0.98	0.93 (0.65; 1.34)	0.89	Amino Acid	Histidine Metabolism
1-oleoyl-2-docosahexaenoyl-GPC (18:1/22:6)*	0.70 (0.52; 0.95)	0.25	0.73 (0.53; 1.02)	0.84	0.73 (0.52; 1.02)	0.89	Lipid	Phosphatidylcholine (PC)
valine	1.39 (1.05; 1.84)	0.25	1.22 (0.88; 1.69)	0.94	1.15 (0.83; 1.60)	0.89	Amino Acid	Leucine, Isoleucine and Valine Metabolism
tyrosine	1.38 (1.02; 1.87)	0.28	1.17 (0.84; 1.64)	0.94	1.17 (0.83; 1.66)	0.89	Amino Acid	Tyrosine metabolism
2-hydroxybutyrate/2-hydroxyisobutyrate	1.41 (1.03; 1.92)	0.25	1.31 (0.93; 1.83)	0.94	1.27 (0.90; 1.81)	0.89	Amino Acid	Glutathione Metabolism
3-(4-hydroxyphenyl) lactate (HPLA)	1.43 (1.04; 1.95)	0.25	1.24 (0.88; 1.75)	0.94	1.21 (0.86; 1.70)	0.89	Amino Acid	Tyrosine Metabolism
2-aminoadipate	1.38 (1.02; 1.87)	0.28	1.16 (0.86; 1.58)	0.94	1.08 (0.78; 1.49)	0.89	Amino Acid	Lysine Metabolism
alpha-hydroxyisocaproate	1.43 (1.03; 1.97)	0.25	1.40 (0.96; 2.04)	0.84	1.40 (0.95; 2.05)	0.89	Amino Acid	Leucine, Isoleucine and Valine Metabolism
imidazole lactate	0.79 (0.58; 1.06)	0.62	0.87 (0.63; 1.19)	0.94	0.86 (0.63; 1.18)	0.89	Amino Acid	Histidine Metabolism
docosadienoate (22:2n6)	1.02 (0.76; 1.37)	0.91	1.07 (0.76; 1.50)	0.94	1.07 (0.77; 1.48)	0.89	Lipid	Polyunsaturated Fatty Acid (n3 and n6)
isovalerylglycine	0.90 (0.68; 1.18)	0.82	0.91 (0.69; 1.20)	0.94	0.93 (0.70; 1.23)	0.89	Amino Acid	Leucine, Isoleucine and Valine Metabolism
glycine	0.81 (0.59; 1.12)	0.71	0.87 (0.62; 1.24)	0.94	0.92 (0.65; 1.30)	0.89	Amino Acid	Glycine, Serine and Threonine Metabolism
sphingomyelin (d18:1/22:2, d18:2/22:1, d16:1/24:2)*	1.08 (0.79; 1.48)	0.84	1.04 (0.76; 1.44)	0.94	1.19 (0.85; 1.67)	0.89	Lipid	Sphingolipid Metabolism
betaine	0.85 (0.62; 1.15)	0.71	0.86 (0.61; 1.21)	0.94	0.85 (0.60; 1.20)	0.89	Amino Acid	Glycine, Serine and Threonine Metabolism

indolelactate	1.21 (0.89; 1.65)	0.71	1.21 (0.87; 1.69)	0.94	1.19 (0.85; 1.66)	0.89	Amino Acid	Tryptophan Metabolism
2-linoleoylglycerol (18:2)	1.09 (0.81; 1.46)	0.84	1.22 (0.89; 1.67)	0.94	1.19 (0.87; 1.63)	0.89	Lipid	Monoacylglycerol
trimethylamine N-oxide	1.13 (0.86; 1.49)	0.75	1.12 (0.83; 1.51)	0.94	1.07 (0.78; 1.45)	0.89	Lipid	Phospholipid Metabolism
hydantoin-5-propionate	1.18 (0.88; 1.59)	0.71	0.99 (0.73; 1.36)	0.99	0.93 (0.68; 1.29)	0.89	Amino Acid	Histidine Metabolism
sphingomyelin (d18:2/23:0, d18:1/23:1, d17:1/24:1)*	1.03 (0.76; 1.39)	0.89	1.14 (0.83; 1.57)	0.94	1.13 (0.82; 1.55)	0.89	Lipid	Sphingolipid Metabolism
sphingomyelin (d18:2/23:1)*	1.16 (0.84; 1.60)	0.75	1.14 (0.82; 1.58)	0.94	1.22 (0.86; 1.72)	0.89	Lipid	Sphingolipid Metabolism
sphingomyelin (d18:2/21:0, d16:2/23:0)*	1.22 (0.88; 1.68)	0.71	1.18 (0.83; 1.66)	0.94	1.19 (0.83; 1.70)	0.89	Lipid	Sphingolipid Metabolism
docosahexaenoylcholine	0.83 (0.62; 1.11)	0.71	0.86 (0.63; 1.17)	0.94	0.81 (0.59; 1.11)	0.89	Lipid	Fatty Acid Metabolism (Acyl Choline)
arachidonoylcholine	0.84 (0.61; 1.15)	0.72	0.93 (0.68; 1.27)	0.94	0.87 (0.63; 1.20)	0.89	Lipid	Fatty Acid Metabolism (Acyl Choline)
eicosenoate (20:1n9 or 1n11)	0.92 (0.69; 1.23)	0.84	0.93 (0.67; 1.28)	0.94	0.90 (0.65; 1.25)	0.89	Lipid	Long Chain Fatty Acid
1-palmitoleoyl-GPC* (16:1)*	0.98 (0.73; 1.32)	0.91	1.10 (0.82; 1.48)	0.94	1.08 (0.80; 1.45)	0.89	Lipid	Lysophospholipid
N6-acetyllysine	1.02 (0.73; 1.41)	0.93	0.94 (0.66; 1.34)	0.94	0.85 (0.59; 1.27)	0.89	Amino Acid	Lysine Metabolism
gamma-glutamylglycine	0.86 (0.64; 1.16)	0.72	0.91 (0.67; 1.24)	0.94	0.93 (0.68; 1.27)	0.89	Peptide	Gamma-glutamyl Amino Acid
glycerol 3-phosphate	1.07 (0.81; 1.43)	0.84	1.12 (0.84; 1.49)	0.94	1.10 (0.83; 1.47)	0.89	Lipid	Glycerolipid Metabolism
phenyllactate (PLA)	1.22 (0.87; 1.71)	0.71	1.10 (0.77; 1.57)	0.94	1.09 (0.75; 1.56)	0.89	Amino Acid	Phenylalanine Metabolism
5alpha-androstan-3beta,17alpha-diol disulfate	0.90 (0.65; 1.24)	0.84	0.95 (0.68; 1.32)	0.94	0.92 (0.66; 1.30)	0.89	Lipid	Androgenic Steroids
N-acetylputrescine	1.20 (0.89; 1.62)	0.71	1.22 (0.89; 1.67)	0.94	1.22 (0.88; 1.68)	0.89	Amino Acid	Polyamine Metabolism
phosphate	1.03 (0.77; 1.39)	0.88	1.14 (0.85; 1.54)	0.94	1.17 (0.86; 1.58)	0.89	Energy	Oxidative Phosphorylation

1-palmitoleoyl-2-linolenoyl-GPC (16:1/18	1.08 (0.80; 1.45)	0.84	1.21 (0.89; 1.64)	0.94	1.16 (0.85; 1.57)	0.89	Lipid	Phosphatidylcholine (PC)
phenylpyruvate	1.21 (0.89; 1.65)	0.71	1.17 (0.85; 1.60)	0.94	1.12 (0.82; 1.54)	0.89	Amino Acid	Phenylalanine Metabolism
glycosyl-N-behenoyl-sphingadienine (d18:2/22:0)*	0.86 (0.63; 1.17)	0.72	1.01 (0.73; 1.40)	0.99	1.12 (0.80; 1.58)	0.89	Lipid	Ceramides
linoleoylcholine*	0.87 (0.64; 1.18)	0.75	0.93 (0.68; 1.27)	0.94	0.92 (0.67; 1.25)	0.89	Lipid	Fatty Acid Metabolism (Acyl Choline)
stearoylcholine*	0.89 (0.66; 1.21)	0.83	0.95 (0.70; 1.29)	0.94	0.90 (0.66; 1.23)	0.89	Lipid	Fatty Acid Metabolism (Acyl Choline)
sphingomyelin (d17:2/16:0, d18:2/15:0)*	1.16 (0.86; 1.58)	0.72	1.12 (0.81; 1.56)	0.94	1.09 (0.78; 1.52)	0.89	Lipid	Sphingolipid Metabolism
phenylalanine	1.42 (1.04; 1.94)	0.25	1.22 (0.87; 1.71)	0.94	1.23 (0.87; 1.74)	0.89	Amino Acid	Phenylalanine metabolism
carnitine	0.84 (0.63; 1.13)	0.71	0.88 (0.64; 1.21)	0.94	0.80 (0.57; 1.11)	0.89	Lipid	Carnitine metabolism
fumarate	1.00 (0.73; 1.37)	0.99	0.92 (0.65; 1.30)	0.94	0.92 (0.65; 1.29)	0.89	Energy	TCA Cycle
palmitoylcholine	0.85 (0.62; 1.15)	0.72	0.93 (0.68; 1.25)	0.94	0.90 (0.66; 1.21)	0.89	Lipid	Fatty Acid Metabolism (Acyl Choline)
kynurenate	1.25 (0.93; 1.69)	0.65	1.11 (0.80; 1.53)	0.94	1.08 (0.78; 1.50)	0.89	Amino Acid	Tryptophan Metabolism
sphingomyelin (d18:1/18:1, d18:2/18:0)	1.38 (0.98; 1.93)	0.39	1.37 (0.97; 1.93)	0.84	1.37 (0.96; 1.94)	0.89	Lipid	Sphingolipid Metabolism
eicosenoylcarnitine (C20:1)*	0.69 (0.52; 0.94)	0.55	0.79 (0.57; 1.08)	0.94	0.76 (0.55; 1.05)	0.89	Lipid	Fatty Acid Metabolism (Acyl Carnitine)
sphingomyelin (d18:2/16:0, d18:1/16:1)*	1.11 (0.82; 1.52)	0.83	1.06 (0.75; 1.49)	0.94	1.09 (0.78; 1.52)	0.89	Lipid	Sphingolipid Metabolism
N-acetylkynurenine (2)	1.19 (0.88; 1.62)	0.71	1.16 (0.84; 1.62)	0.94	1.19 (0.85; 1.65)	0.89	Amino Acid	Tryptophan Metabolism
1,5-anhydroglucitol (1,5-AG)	0.88 (0.62; 1.26)	0.83	0.97 (0.67; 1.39)	0.95	0.92 (0.64; 1.33)	0.89	Carbohydrate	Glycolysis, Gluconeogenesis, and Pyruvate Metabolism

citrulline	0.93 (0.67; 1.28)	0.84	0.96 (0.69; 1.34)	0.94	0.88 (0.63; 1.23)	0.89	Amino Acid	Urea Cycle; Arginine and Proline Metabolism
pyroglutamine*	0.91 (0.62; 1.34)	0.84	0.94 (0.64; 1.39)	0.94	0.89 (0.59; 1.33)	0.89	Amino Acid	Glutamate Metabolism
picolinate	1.23 (0.90; 1.69)	0.71	1.21 (0.87; 1.68)	0.94	1.22 (0.89; 1.69)	0.89	Amino Acid	Tryptophan Metabolism
sphingomyelin (d18:2/14:0, d18:1/14:1)*	1.20 (0.85; 1.68)	0.72	1.13 (0.80; 1.59)	0.94	1.08 (0.76; 1.53)	0.89	Lipid	Sphingolipid Metabolism
dihomo-linoleoylcarnitine (C20:2)*	0.77 (0.56; 1.05)	0.55	0.82 (0.59; 1.14)	0.94	0.81 (0.58; 1.12)	0.89	Lipid	Fatty Acid Metabolism (Acyl Carnitine)
1-methyladenosine	1.32 (0.97; 1.78)	0.43	1.22 (0.88; 1.68)	0.94	1.19 (0.86; 1.65)	0.89	Nucleotide	Purine Metabolism, Adenine containing
aspartate	1.25 (0.93; 1.70)	0.65	1.12 (0.82; 1.53)	0.94	1.07 (0.78; 1.46)	0.89	Amino Acid	Alanine and Aspartate Metabolism
urea	0.95 (0.70; 1.27)	0.84	0.89 (0.65; 1.22)	0.94	0.86 (0.63; 1.17)	0.89	Amino Acid	Urea cycle; Arginine and Proline Metabolism
3-hydroxydecanoate	1.14 (0.84; 1.55)	0.75	1.11 (0.80; 1.55)	0.94	1.14 (0.81; 1.59)	0.89	Lipid	Fatty Acid, Monohydroxy
beta-citrylglutamate	1.32 (0.99; 1.77)	0.39	1.26 (0.92; 1.71)	0.94	1.19 (0.87; 1.63)	0.89	Amino Acid	Glutamate Metabolism
mannitol/sorbitol	1.02 (0.75; 1.40)	0.91	0.96 (0.68; 1.35)	0.94	0.90 (0.64; 1.26)	0.89	Carbohydrate	Fructose, Mannose and Galactose Metabolism
sphingomyelin (d17:1/16:0, d18:1/15:0, d16:1/17:0)*	1.14 (0.85; 1.54)	0.75	1.11 (0.80; 1.55)	0.94	1.20 (0.85; 1.69)	0.89	Lipid	Sphingolipid Metabolism
choline	1.06 (0.78; 1.44)	0.84	0.93 (0.67; 1.28)	0.94	0.90 (0.64; 1.25)	0.89	Lipid	Phospholipid Metabolism
6-oxopiperidine-2-carboxylate	0.93 (0.69; 1.25)	0.84	0.85 (0.62; 1.15)	0.94	0.82 (0.60; 1.12)	0.89	Amino Acid	Lysine Metabolism
L-urobilin	0.92 (0.65; 1.29)	0.84	0.91 (0.64; 1.29)	0.94	0.93 (0.65; 1.33)	0.89	Cofactors and Vitamins	Hemoglobin and Porphyrin Metabolism

4-methoxyphenol sulfate	1.04 (0.78; 1.39)	0.87	1.06 (0.80; 1.42)	0.94	1.08 (0.81; 1.45)	0.89	Amino Acid	Tyrosine Metabolism
guanidinoacetate	0.83 (0.58; 1.19)	0.72	0.91 (0.63; 1.32)	0.94	0.92 (0.63; 1.35)	0.89	Amino Acid	Creatine Metabolism
p-cresol glucuronide*	1.08 (0.80; 1.46)	0.84	1.09 (0.79; 1.49)	0.94	1.09 (0.79; 1.50)	0.89	Amino Acid	Tyrosine Metabolism
sphingomyelin (d18:1/19:0, d19:1/18:0)*	1.90 (0.88; 1.60)	0.72	1.23 (0.90; 1.70)	0.94	1.23 (0.88; 1.72)	0.89	Lipid	Sphingolipid Metabolism
phenylacetate	1.12 (0.83; 1.51)	0.83	1.08 (0.78; 1.49)	0.94	1.13 (0.81; 1.57)	0.89	Amino Acid	Phenylalanine Metabolism
N6,N6,N6-trimethyllysine	1.11 (0.80; 1.56)	0.84	0.94 (0.64; 1.38)	0.94	0.94 (0.64; 1.38)	0.90	Amino Acid	Lysine Metabolism
beta-hydroxyisovalerate	1.08 (0.80; 1.47)	0.84	1.06 (0.77; 1.46)	0.94	1.06 (0.76; 1.46)	0.90	Amino Acid	Leucine, Isoleucine and Valine Metabolism
phenylacetylglutamine	1.10 (0.81; 1.51)	0.84	1.04 (0.76; 1.44)	0.94	1.06 (0.76; 1.47)	0.90	Peptide	Acetylated Peptides
indole-3-carboxylate	1.09 (0.81; 1.47)	0.84	1.06 (0.79; 1.41)	0.94	1.06 (0.77; 1.45)	0.90	Amino Acid	Tryptophan Metabolism
alpha-hydroxyisovalerate	0.94 (0.70; 1.27)	0.84	0.94 (0.67; 1.32)	0.94	0.95 (0.68; 1.33)	0.91	Amino Acid	Leucine, Isoleucine and Valine Metabolism
7-methylguanine	1.12 (0.81; 1.55)	0.83	1.04 (0.75; 1.45)	0.94	1.05 (0.75; 1.48)	0.91	Nucleotide	Purine Metabolism, Guanine containing
2-palmitoleoyl-GPC* (16:1)*	0.96 (0.72; 1.28)	0.87	1.05 (0.78; 1.42)	0.94	1.03 (0.76; 1.39)	0.92	Lipid	Lysophospholipid
3-methylglutaconate	1.05 (0.78; 1.42)	0.84	1.06 (0.78; 1.46)	0.94	1.02 (0.74; 1.41)	0.92	Amino Acid	Leucine, Isoleucine and Valine Metabolism
sphingomyelin (d18:1/22:1, d18:2/22:0, d16:1/24:1)*	0.96 (0.72; 1.30)	0.87	1.01 (0.71; 1.43)	0.99	1.03 (0.73; 1.46)	0.92	Lipid	Sphingolipid Metabolism
oleate/vaccenate (18:1)	1.08 (0.80; 1.64)	0.84	1.05 (0.75; 1.46)	0.94	1.04 (0.74; 1.45)	0.92	Lipid	Long Chain Fatty Acid
1,2-dipalmitoyl-GPC (16:0/16:0)	0.95 (0.70; 1.29)	0.84	0.99 (0.71; 1.38)	0.99	0.98 (0.70; 1.38)	0.92	Lipid	Phosphatidylcholine (PC)
5alpha-androstan-3alpha,17beta-diol disulfate	0.88 (0.62; 1.25)	0.83	0.93 (0.64; 1.34)	0.94	0.95 (0.65; 1.39)	0.92	Lipid	Androgenic Steroids

N6-carbamoylthreonyladenosine	1.11 (0.82; 1.52)	0.83	1.00 (0.72; 1.38)	0.99	0.96 (0.69; 1.33)	0.92	Nucleotide	Purine Metabolism, Adenine containing
maltose	1.13 (0.86; 1.49)	0.75	1.02 (0.76; 1.36)	0.98	0.97 (0.73; 1.30)	0.92	Carbohydrate	Glycogen Metabolism
phenylacetylglutamate	1.06 (0.78; 1.43)	0.84	1.02 (0.75; 1.39)	0.97	1.02 (0.74; 1.41)	0.92	Peptide	Acetylated Peptides
creatinine	1.04 (0.72; 1.49)	0.89	1.00 (0.68; 1.48)	0.99	0.95 (0.64; 1.42)	0.92	Amino Acid	Creatine Metabolism
N-acetyltaurine	1.07 (0.77; 1.50)	0.84	1.13 (0.77; 1.64)	0.94	1.02 (0.69; 1.52)	0.92	Amino Acid	Methionine, Cysteine, SAM and Taurine Metabolism
3-methylcytidine	1.07 (0.79; 1.46)	0.84	1.07 (0.78; 1.46)	0.94	1.03 (0.76; 1.40)	0.92	Nucleotide	Pyrimidine Metabolism, Cytidine containing
3-hydroxybutyrate (BHBA)	0.95 (0.72; 1.26)	0.84	0.99 (0.74; 1.33)	0.99	0.98 (0.73; 1.32)	0.92	Lipid	Ketone Bodies
3-hydroxy-3-methylglutarate	1.17 (0.85; 1.61)	0.72	1.04 (0.73; 1.47)	0.95	0.96 (0.67; 1.39)	0.92	Lipid	Mevalonate Metabolism
isobutyrylglycine (C4)	0.95 (0.71; 1.27)	0.84	0.96 (0.70; 1.31)	0.94	0.98 (0.71; 1.35)	0.92	Amino Acid	Leucine, Isoleucine and Valine Metabolism
N-acetylglucosaminylasparagine	1.16 (0.86; 1.55)	0.72	1.05 (0.77; 1.42)	0.94	1.01 (0.74; 1.38)	0.95	Carbohydrate	Aminosugar Metabolism

Model 1: Poisson regression models included the individual metabolites, age and sex. Model 2: Model 1 + smoking, education, family history of diabetes, METs of physical activity (physical activity score in BPRHS), waist circumference (cm), BMI (kg/m^2), and alcohol consumption (g/day). Model 3: Model 2 + use of antihypertensive medications and statins or other lipid lowering medications. Benjamini-Hochberg FDR p-values were calculated for 104 metabolites.

Supplemental Table S5. Incidence Rate Ratios (95% confidence intervals) for progression from prediabetes to type 2 diabetes, according to 1 standard deviation change in each metabolite, for select metabolites in the San Juan Overweight Adult Longitudinal Study (SOALS) and Boston Puerto Rican Health Study (BPRHS) cohorts.

Metabolite	Model 1 IRR (95% CI)	FDR p-value	Model 2 IRR (95% CI)	FDR p-value	Model 3 IRR (95% CI)	FDR p-value	SUPER-PATHWAY	SUB-PATHWAY
**SOALS**								
5-oxoproline	1.55 (1.19; 2.03)	0.04	1.61 (1.22; 2.13)	0.03	1.62 (1.22; 2.15)	0.03	Amino Acid	Glutathione Metabolism
3-methyl-2-oxovalerate	1.66 (1.19; 2.33)	0.05	1.83 (1.28; 2.61)	0.03	1.83 (1.28; 2.61)	0.03	Amino Acid	Leucine, Isoleucine and Valine Metabolism

2-hydroxy-3-methyl-valerate	1.80 (1.28; 2.52)	0.04	1.78 (1.27; 2.50)	0.03	1.78 (1.26; 2.50)	0.03	Amino Acid	Leucine, Isoleucine and Valine Metabolism
3-methyl-2-oxobutyr-ate	1.60 (1.19; 2.13)	0.04	1.66 (1.22; 2.24)	0.03	1.66 (1.22; 2.25)	0.03	Amino Acid	Leucine, Isoleucine and Valine Metabolism
ornithine	1.46 (1.12; 1.90)	0.07	1.52 (1.15; 2.00)	0.04	1.54 (1.17; 2.04)	0.05	Amino Acid	Urea cycle; Arginine and Pro-line Metabolism
4-methyl-2-oxopenta-noate	1.61 (1.15; 2.26)	0.07	1.71 (1.20; 2.43)	0.04	1.71 (1.20; 2.44)	0.05	Amino Acid	Leucine, Isoleucine and Valine Metabolism
1-palmitoyl-2-pal-mitoleoyl-GPC (16:0/16:1)*	1.51 (1.18; 1.95)	0.04	1.47 (1.14; 1.90)	0.04	1.48 (1.14; 1.91)	0.05	Lipid	Phosphatidylcholine (PC)
**BPRHS**								
5-oxoproline	0.98 (0.73; 1.32)	0.97	0.94 (0.69; 1.27)	0.99	0.91 (0.66; 1.26)	0.98	Amino Acid	Glutathione Metabolism
3-methyl-2-oxova-lerate	1.60 (1.17; 2.18)	0.20	1.6 (1.13; 2.26)	0.78	1.49 (1.05; 2.1)	0.98	Amino Acid	Leucine, Isoleucine and Valine Metabolism
2-hydroxy-3-methyl-valerate	1.23 (0.9; 1.67)	0.84	1.15 (0.82; 1.63)	0.99	1.14 (0.80, 1.61)	0.98	Amino Acid	Leucine, Isoleucine and Valine Metabolism
3-methyl-2-oxobutyr-ate	1.49 (1.1; 2.03)	0.26	1.36 (1; 1.86)	0.90	1.28 (0.95; 1.74)	0.98	Amino Acid	Leucine, Isoleucine and Valine Metabolism
ornithine	0.91 (0.65; 1.29)	0.90	0.63 (0.43; 0.92)	0.86	0.60 (0.40, 0.90)	0.98	Amino Acid	Urea cycle; Arginine and Pro-line Metabolism
4-methyl-2-oxopenta-noate	1.42 (1.03; 1.97)	0.44	1.44 (1.01; 2.06)	0.86	1.34 (0.94, 1.92)	0.98	Amino Acid	Leucine, Isoleucine and Valine Metabolism
1-palmitoyl-2-pal-mitoleoyl-GPC (16:0/16:1)*	1.23 (0.9; 1.67)	0.84	1.18 (0.86; 1.63)	0.99	1.08 (0.77, 1.51)	0.98	Lipid	Phosphatidylcholine (PC)

Model 1: Poisson regression models included the individual metabolites, age, and sex. Model 2: Model 1 + smoking, education, METs of physical activity (physical activity score in BPRHS), waist circumference, BMI (kg/m^2), and alcohol consumption (g/day). Model 3: Model 2 + use of anti-hypertensive medications and statins or other lipid lowering medications.Benjamini-Hochberg FDR p-values were calculated for 104 metabolites.
